# Phase II study comparing nasal pressure monitoring with capnography during invasive endoscopic procedures: a single-center, single-arm trial

**DOI:** 10.1038/s41598-023-28213-y

**Published:** 2023-01-23

**Authors:** Hiroki Nagashima, Rintaro Mikata, Shiroh Isono, Sadahisa Ogasawara, Harutoshi Sugiyama, Izumi Ohno, Shin Yasui, Tomoaki Matsumura, Keisuke Koroki, Yuko Kusakabe, Yoshifumi Miura, Motoyasu Kan, Shikiko Maruta, Toshihito Yamada, Ryo Takemura, Yasunori Sato, Jun Kato, Naoya Kato

**Affiliations:** 1 Department of Gastroenterology, Graduate School of Medicine, Chiba University, Inohana 1-8-1, Chiba, 260-8670 Japan; 2 Department of Anesthesiology, Graduate School of Medicine, Chiba University, Chiba, Japan; 3 Translational Research and Development Center, Chiba University Hospital, Chiba, Japan; 4 Clinical and Translational Research Center, Keio University Hospital, Tokyo, Japan; 5 Department of Preventive Medicine and Public Health, Keio University School of Medicine, Tokyo, Japan

**Keywords:** Gastroenterology, Medical research

## Abstract

Nasal pressure signal is commonly used to evaluate obstructive sleep apnea. This study aimed to assess its safety for respiratory monitoring during sedation. A total of 45 adult patients undergoing sedation with propofol and fentanyl for invasive endoscopic procedures were enrolled. While both nasal pressure and capnograph signals were continuously recorded, only the nasal pressure signal was displayed. The primary outcome was the incidence of oxygen desaturation below 90%. The secondary outcomes were the ability to predict the desaturation and incidence of harmful events and false alarms, defined as an apnea waveform lasting more than 3 min without desaturation. Of the 45 participants, 43 completed the study. At least one desaturation event occurred in 12 patients (27.9%; 95% confidence interval 15.3–43.7%). In these 12 patients, more than half of the desaturation events were predictable in 9 patients by capnography and 11 patients by nasal pressure monitoring (*p* = 0.59). In the 43 patients, false alarms were detected in 7 patients with capnography and 11 patients with nasal pressure monitoring (*p* = 0.427). Harmful events unrelated to nasal pressure monitoring occurred in 2 patients. Nasal pressure monitoring is safe and possibly useful for respiratory monitoring despite false alarms during sedation.

## Introduction

The upper interventional endoscopy procedures of endoscopic submucosal dissection (ESD), endoscopic retrograde cholangiopancreatography (ERCP), endoscopic ultrasound-guided fine-needle aspiration (EUS-FNA), and double-balloon enteroscopy (DBE) are currently performed under sedation with sedative agents, including propofol, dexmedetomidine, and benzodiazepine, and analgesics, including fentanyl, pentazocine, and pethidine, to reduce patient distress and achieve successful completion. However, hypoxemia associated with respiratory depression or hemodynamic instability is well known to occur frequently during sedation. Furthermore, cardiopulmonary events during endoscopy are described to be the most important fatal complications^1^. Even tracheal intubation (0.09%) and death (0.03%) have occurred in some cases where propofol was used for sedation in endoscopy^2^. Therefore, appropriate use of cardiorespiratory monitoring is recommended during and after endoscopy with sedation.

The European Society of Gastrointestinal Endoscopy recommended that capnography be considered during the administration of propofol in specific situations, including high-risk patients, intended deep sedation, and long procedure duration^3^. Furthermore, the American Society for Gastrointestinal Endoscopy suggested that capnography be considered for patients undergoing endoscopy targeting deep sedation^4^. Therefore, capnography during an endoscopic procedure with deep sedation has been recommended in Japan^5^. However, capnography does not precisely characterize the nature of breathing because end-tidal carbon dioxide is an indirect and lagging indicator of respiratory status rather than a direct measurement of changes in respiratory volume^6,7^. Clinical trials comparing capnography with standard monitoring have reported controversial results^8–17^. Therefore, the use of capnography has not been established as a recognized standard for respiratory monitoring due to other factors, including cost, false alarms, and sensitivity to respiratory abnormalities^16–18^. A recent study revealed that approximately 50% of episodes with oxygen desaturation or hypoxemia occurred in the absence of capnography-observed apnea-altered ventilation^16^.

A nasal pressure monitor is a simple and easy-to-use tool for assessing airflow and is commonly used during polysomnography, whereas a pneumotachograph, the gold standard method for measuring airflow, is inconvenient for use in daily clinical practice due to the requirement for a well-fitting mask^19,20^. The waveform of a nasal pressure monitor is comparable in shape and amplitude to that of a pneumotachograph and provides a semiquantitative estimation of airflow^21^. Hypopnea can be judged more easily using this monitoring method because its wave amplitude changes dynamically and can be interpreted as changes in breathing depth. Furthermore, the use of a nasal pressure monitor has been reported to be useful for detecting airway obstruction, and a characteristic flattened waveform is indicative of upper airway resistance and inspiratory flow limitation^19^.

For these reasons, a nasal pressure monitor can distinguish between the central respiratory disorder due to drug overdose and the airway obstruction that requires management by its waveform^19–21^. Additionally, nasal pressure monitoring is recommended over capnography by the American Academy of Sleep Medicine for sleep apnea testing^19^. It has been also reported to detect respiratory abnormalities with increased sensitivity and earlier than pulse oximetry in a previous study^22^. Thus, we hypothesized that the nasal pressure monitor could provide an early warning for appropriate interventions, thereby preventing hypoxemia during sedation. If this hypothesis is correct, a nasal pressure monitor should be non-inferior to capnography in preventing the incidence of hypoxemic events, defined as a decrease in peripheral capillary oxygen saturation (SpO_2_) below 90%, during sedation. Thus, a prospective, randomized, non-inferiority, phase II and phase III trial was designed to test this hypothesis. This phase II trial aimed to ensure that the nasal pressure monitor can be used as a respiratory monitor during invasive endoscopic procedures under sedation with propofol and that there are no safety concerns. The waveforms of the nasal pressure monitor and capnography were also compared to help understand their characteristics.

## Materials and methods

### Study population

Patients scheduled for ERCP, ESD, EUS-FNA, or DBE under sedation at Chiba University Hospital between December 2019 and April 2020 were enrolled. The inclusion criteria included age ≥ 20 years, SpO_2_ ≥ 95% on room air, systolic blood pressure ≥ 90 mmHg, and ability to provide written informed consent. The exclusion criteria included regular use of opioids or benzodiazepines for purposes other than sleeping; participation in another clinical trial within 4 weeks of registering for this trial; drug abuse or a physical, mental, or social condition that made enrollment or evaluation impossible; severe cardiac disorder; heart rate < 50 bpm; ASA physical status classification IV or V; severe thyroid disease; pregnancy or lactation; possibility of allergic reaction to propofol or fentanyl; and any other condition that could impair the patient’s safety or might limit the ability of the patient to comply with the protocol.

All cases were registered centrally at the data center (Chiba University Hospital). All data were collected using case report forms (CRFs), and all CRFs were submitted to the data center. The data center used a system with which it was possible to acquire access logs and validation, enter the CRF data, and create data sets. The database was locked after verification by the principal investigator. This study was approved by the Research Ethics Committee of the Graduate School of Medicine, Chiba University (approval number 3180015). This trial was registered in the Japan Registry of Clinical Trials (identifier jRCTs032190146) on November 29, 2019. All methods were performed in accordance with the relevant guidelines and regulations of the Declaration of Helsinki. Written informed consent was obtained from all participants.

### Study design

This was a prospective, single-center, single-arm phase II trial to confirm the safety of the nasal pressure monitor as a novel approach to monitoring during invasive endoscopic procedures with non-anesthesiologist administration of propofol and fentanyl sedation. All patients were placed in the treatment position after both the capnography sensor (CO_2_ Sensor Kit TG-920P; Nihon Kohden Corp., Tokyo, Japan) and the intranasal pressure sensor (Nihon Kohden Corp., Tokyo, Japan) were attached to the nose by the sedation doctor. Before sedation, the capnography waveform was configured not to be displayed on the monitor screen (Bedside Monitor CSM-01901; Nihon Kohden Corp., Tokyo, Japan), so only the nasal pressure monitor was used as a respiratory monitor. The waveforms were continuously recorded with the monitoring device for later playback and review.

### Procedure (intervention)

All patients underwent standard monitoring with continuous pulse oximetry. Blood pressure was monitored every 2.5 min for the first 15 min and then every 5 min, and clinical observation, including the Ramsay Sedation Scale, was performed every 5 min with electrocardiogram monitoring. Bispectral Index waveforms were also continuously recorded on the monitor. The information recorded on the monitor was later reviewed.

The patients were sedated with propofol and fentanyl by an anesthesiologist-trained endoscopist, and the drug amount was appropriately adjusted according to the protocol for sedation during an invasive endoscopic procedure developed by the Anesthesiology Department of our hospital. When oxygen desaturation of SpO_2_ < 95% or apnea ≥ 20 s occurred, interventions to restore ventilation and/or oxygenation were performed by the endoscopic team initiated by the physician performing the sedation. The interventions were as follows: increasing oxygen supplementation, patient stimulation, a chin-lift or jaw-thrust maneuver, withholding medication, and insertion of the nasopharyngeal tube. When oxygen desaturation of SpO_2_ < 95% or < 90% occurred, the existence of respiratory abnormalities and the type of respiratory abnormality were recorded as apnea; hypopnea, defined as ≥ 50% reduction from the baseline waveform lasting 10 s; decreased respiratory rate, defined as a respiratory rate < 8 breaths/min; and obstructive respiratory abnormality. If SpO_2_ decreased to < 95% and then quickly dropped to < 90%, the event was recorded as SpO_2_ < 90%.

All adverse events that occurred on day 1 after treatment were documented, and all serious adverse events within 1 week were also recorded, in addition to adverse events suspected of being related to the testing device.

Following the procedure, the monitor records for both capnography and the nasal pressure monitor were reviewed by three physicians, including an expert on waveform analysis for the nasal pressure monitor. The incidence of oxygen desaturation below 95% or 90% was evaluated and compared with the existence of respiratory abnormalities and the type of respiratory abnormality based on the waveforms of the nasal pressure monitor and capnography at the point at which oxygen desaturation occurred. The respiratory abnormalities that occurred within 1 min before hypoxemic events were considered with reference to a previous report in which hypoxemic events could be delayed by 45–60 s after an apnea episode in patients with obstructive sleep apnea^23^. Additionally, data on false alarms, defined as events in which oxygen desaturation did not occur despite the waveform indicating that apnea lasted > 3 min, were collected.

### Study outcomes

The primary outcome was the incidence of desaturation events, defined as a fall of SpO_2_ below 90% for any time in seconds during the procedure. The secondary outcome was the incidence of adverse events. A comparison between capnography and nasal pressure waveforms was also performed to understand the characteristics of each monitor and to see if there were problems. The respiratory waveforms were specifically analyzed when SpO_2_ dropped to < 90%. If any respiratory abnormality on the waveform was detected before the desaturation event as the cause of desaturation, we considered that the monitor accurately detected the cause of desaturation. An accurate detection rate was calculated for each patient who had desaturation events. For example, when SpO_2_ dropped to < 90% eight times and the respiratory monitor detected respiratory abnormalities at least four times before the SpO_2_ dropped to < 90%, the accurate detection rate was considered ≥ 50%. Then, the percentage of patients with an accurate detection rate of ≥ 50% for the number of patients who had desaturation events was determined as respiratory abnormality detectability for each monitoring device. This method was chosen because the number of desaturation events greatly varied between patients. If all events were treated the same, the outcome would be influenced by similar events involving the same patients. A detection rate of ≥ 50% was adopted according to a previous report in which apnea-altered ventilation could not be detected in approximately 50% of cases with capnography before the hypoxemic event^12^. Respiratory abnormalities included apnea > 20 s, hypopnea, respiratory rate ≤ 8 breaths/min, and obstructive respiratory disorder. The two monitors were compared in terms of false alarms and respiratory abnormality detectability.

### Statistical analysis

Using propofol-based sedation, the hypoxemia rate during invasive endoscopic procedures under sedation was reported to range from 12 to 50%^16,24–28^. Therefore, in this study, the hypoxemia rate was estimated to be 20% as the expected value and 40% as the threshold value. Given these assumptions, a sample size of 43 cases with a power of 80% and a two-sided significance rate of 5% was required. Taking the deviation into account, a sample size of 45 patients was determined.

Continuous data were expressed as the mean and standard deviation. Categorical data were presented as numbers and percentages. Categorical variables were compared using Fisher’s exact test. Logistic regression analysis was used for univariate analysis, and the OR and 95% CI were calculated. The CI for the percentage of two values was calculated using the Clopper–Pearson interval. A two-tailed p-value < 0.05 was considered statistically significant. For comparison of multiple items, a p-value < 0.05/α (α indicating the number of multiplicities) was considered significant using the Bonferroni correction. When comparing respiratory abnormalities, a p-value < 0.0125 was set as the significance level because there were four variables. All data analyses were performed using SAS (version 9.4, SAS Institute, Cary, NC, USA).

### Ethics statement

This study was approved by the Research Ethics Committee of the Graduate School of Medicine, Chiba University (approval number 3180015). Written informed consent was obtained from all participants.

## Results

### Analysis population

**Figure 1:**
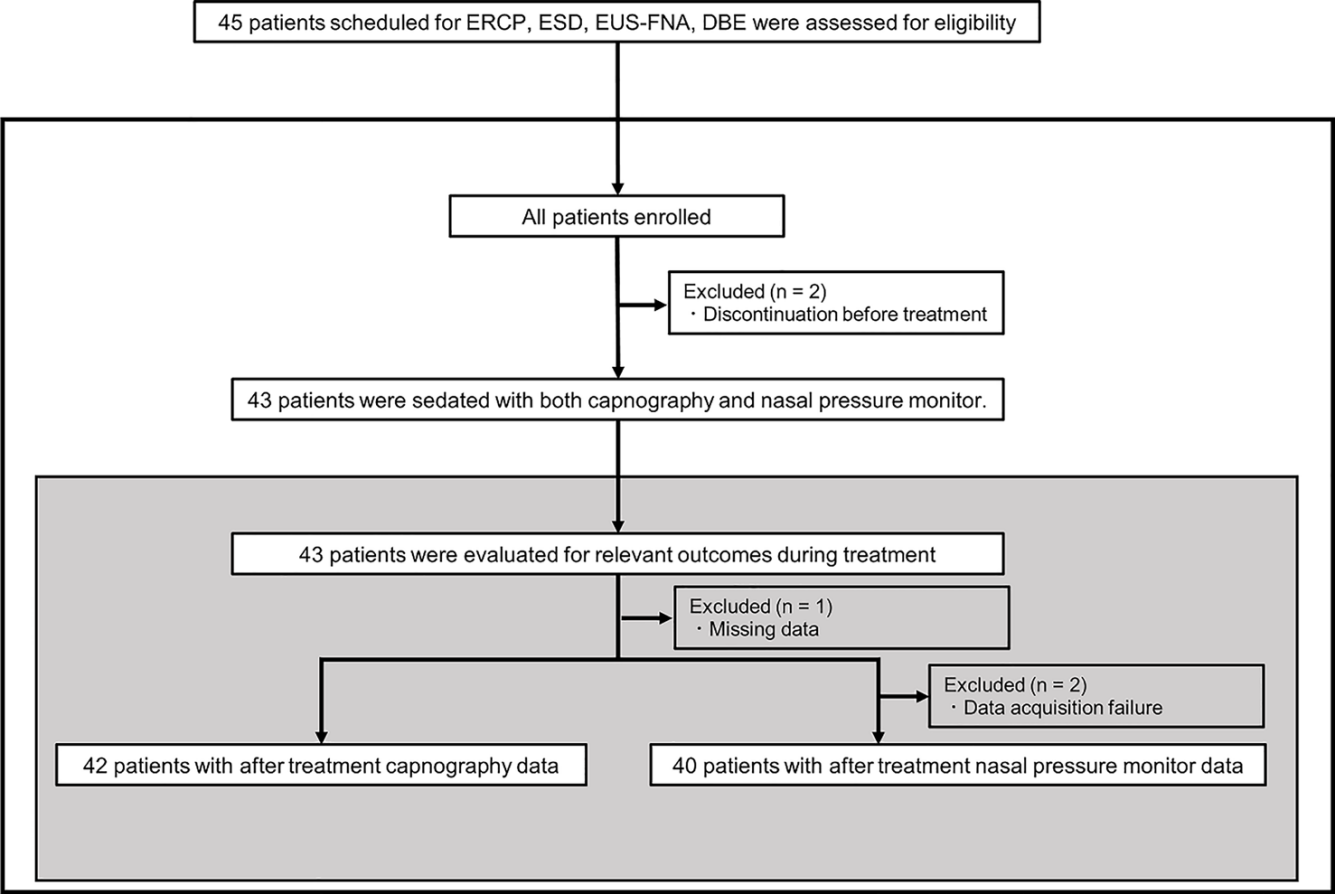
Study population and patient enrollment for analysis.

A total of 45 adult patients were assessed for eligibility, and all the patients were enrolled. Of the 45 patients, two patients dropped out before treatment: one had the procedure canceled, and the other’s respiratory waveform was not displayed from the start due to an equipment malfunction. Thus, we analyzed data from both monitoring waveforms during the treatment of 43 patients. Figure 1 shows the study population and patient enrollment for analysis.

### Patient and procedural characteristics

Among the 45 patients, 29 were males, and 16 were females, aged 28–88 years. For 47 procedures, ESD was performed in 5 patients, ERCP in 28, EUS-FNA in 12, and DBE in 2. For one patient who had both DBE and ERCP and three patients who had both EUS-FNA and ERCP, two procedures were performed in the same operative session. All these procedures were performed in one session with a single continuous sedation. Additional baseline characteristics for the study population are shown in Table 1.

**Table 1:** Patient demographic information and indications for procedure.

Factor	Values
	(*n* = 45)
Sex, male	29 (64.4%)
Age (years), mean ± SD	69 (12.6)
Body mass index (kg/m^2^), mean ± SD	23.4 (4.0)
Smoking (current and previous)	27 (60.0%)
Alcohol abuse	7 (15.6)
Allergies	13 (28.9%)
Past history	
Ischemic heart disease	1 (2.2%)
Hypertension	19 (42.2%)
Dyslipidemia	7 (15.6%)
Diabetes	12 (26.7%)
COPD or asthma	4 (8.9%)
Regular medication	
Sleeping drugs	2 (4.8%)
Antipsychotics	1 (2.4%)
Anxiolytics	0 (0%)
Antihypertensive drugs	19 (45.2%)
ASA class	
I	16 (35.6%)
II	29 (64.4%)
III	0 (0%)
CXR abnormalities	10 (22.2%)
STOP-Bang questionnaire ≥ 3	33 (73.3%)
Mallampati score ≥ 3	25 (55.6%)
Excess neck soft tissue	25 (55.6%)
Baseline systolic blood pressure (mmHg), mean ± SD	129.6 (17.6)
Baseline oxygen saturation (%), mean ± SD	97.4 (1.0)
Baseline heart rate (beats/min), mean ± SD	72 (12.3)
Total propofol dose (mg), mean ± SD	120.7 (68.0)
Total fentanyl dose (μg), mean ± SD	242.3 (139.8)
Procedure time (min), mean ± SD	65.3 (29.4)

Values are presented as *n* (%), unless otherwise specified, values are presented as n (percent). The STOP-Bang questionnaire consists of eight dichotomous (yes/no) questions: (1) snoring, (2) daytime fatigue, (3) observed apnea, (4) high blood pressure, and (5) BMI ≥ 30, (6) age ≥ 50 years, (7) neck circumference ≥ 40 cm, and (8) male sex. The total score ranges from 0 to 8.

*ASA* American Society of Anesthesiologists, *COPD* chronic obstructive pulmonary disease, *CXR* chest X-ray, *SD* standard deviation.

### Primary outcome

Of the 43 patients who underwent endoscopic treatment, 12 patients had oxygen desaturation of SpO_2_ < 90% (27.9%; 95% confidence interval (CI) 15.3–43.7%). The mean time of SpO_2_ < 90% for those 12 patients was 98 s (interquartile range 9–107), and the minimum value of SpO_2_ was 75%. To restore SpO_2_, we performed an increase in oxygen flow rate in 10 cases, a chin-lift or jaw-thrust maneuver in 8 cases, patient stimulation in 6 cases, and drug infusion cessation in 2 cases, while SpO_2_ was restored without any treatment in 2 cases. No patient required nasopharyngeal tube insertion or tracheal intubation and was forced to discontinue endoscopic treatment.

### Adverse events

Table 2 shows the adverse events after treatment. Serious adverse events occurred in two patients: postoperative gastrointestinal bleeding after ESD and aspiration pneumonia after EUS-FNA. Other adverse events occurred in five patients: hyperamylasemia after ERCP in one patient and biliary infections after ERCP in four patients. None of these adverse events occurred due to the use of the nasal pressure monitor.

**Table 2:** Postoperative adverse events.

Factor	Any	95% CI
	(*n* = 43)	
Severe adverse events	2 (4.7%)	0.6–15.8%
Postoperative bleeding	1 (2.3%)	0.1–12.3%
Aspiration pneumonia	1 (2.3%)	0.1–12.3%
The other adverse events	5 (11.6%)	3.9–25.1%
Hyperamylasemia	1 (2.3%)	0.1–12.3%
Biliary infection	4 (9.3%)	2.6–22.1%

A severe adverse event was defined as an event that resulted in death, the possibility of death, the disorders that interfere with daily life, the possibility of the disorders, the postponement of discharge for treatment, serious adverse events as described above, or the possibility of affecting future generations.

*CI* confidence interval.

### Risk factors for oxygen desaturation

Table 3 shows the relationship between oxygen desaturation and patient or procedural characteristics. No significant differences in patient age, sex, body mass index, smoking and alcohol history, past medical history, and regular medication were observed between patients with and without oxygen desaturation of SpO_2_ < 90%. On univariate analysis, allergies (*p* = 0.0234) and American Society of Anesthesiologists (ASA) class I (*p* = 0.0037) were risk factors for oxygen desaturation during treatment. No patient had ASA class ≥ III. Multivariate analysis was not performed due to the small population size in the hypoxic group.

**Table 3:** Univariate analysis of risk factors associated with oxygen desaturation of SpO_2_ < 90%.

Variables	SpO_2_ < 90%	SpO_2_ 190%	*p*	OR (95% CI)
	(*n* = 12)	(*n* = 31)		
Sex, male	8 (66.7%)	20 (64.5%)	0.851	1.15 (0.27–4.99)
Age (years), mean ± SD	68.9 (10.6)	69.1 (13.8)	0.845	1.00 (0.94–1.05)
Body mass index (kg/m2), mean ± SD	24.7 (4.5)	22.9 (3.8)	0.18	1.13 (0.94–1.36)
Endoscopic treatment				
ESD	1 (8.3%)	4 (12.9%)	0.97	–
ERCP	8 (66.7%)	20 (64.5%)	0.846	0.86 (0.18–4.04)
EUS-FNA	5 (41.7%)	7 (22.6%)	0.12	0.30 (0.07–1.37)
DBE	2 (16.7%)	0 (0.0%)	0.976	–
Smoking (current and previous)	7 (58.3%)	20 (64.5%)	0.851	1.15 (0.27–4.99)
Alcohol abuse	0 (0%)	7 (22.6%)	0.954	–
Allergies	6 (50.0%)	6 (19.4%)	0.023	0.17 (0.04–0.79)
Past history				
Ischemic heart disease	0 (0%)	1 (3.2%)	0.983	–
Hypertension	4 (33.3%)	15 (48.4%)	0.585	1.50 (0.35–6.42)
Dyslipidemia	2 (16.7%)	5 (16.1%)	0.81	0.80 (0.13–4.95)
Diabetes	3 (25.0%)	9 (29.0%)	1	1.00 (0.21–4.77)
COPD or Asthma	1 (8.3%)	3 (9.7%)	0.731	0.64 (0.05–7.95)
Regular medication				
Sleeping drug	0 (0%)	2 (6.7%)	0.976	–
Antipsychotic	0 (0%)	1 (3.3%)	0.983	–
Anxiolytic	0 (0%)	0 (0.0%)	–	–
Antihypertensive drug	4 (40.0%)	15 (50.0%)	0.585	1.50 (0.35–6.42)
ASA class				
1	9 (75.0%)	6 (19.4%)	Ref	–
2	3 (25.0%)	25 (80.6%)	0.004	11.66 (2.22–61.26)
3	0 (0%)	0 (0.0%)	–	–
CXR abnormalities	4 (33.3%)	6 (19.4%)	0.515	1.72 (0.34–8.68)
STOP-Bang questionnaire 13	12 (100.0%)	19 (61.3%)	0.938	–
Mallampati score 13	5 (41.7%)	19 (61.3%)	0.581	1.50 (0.36–6.32)
Excess neck soft tissue	8 (66.7%)	16 (51.6%)	0.361	0.49 (0.11–2.26)
Baseline systolic blood pressure (mmHg), mean ± SD	130.5 (19.1)	128.7 (17.6)	0.689	0.99 (0.95–1.03)
Baseline oxygen saturation (%), mean ± SD	97.5 (0.8)	97.4 (1.1)	0.513	1.29 (0.61–2.73)
Baseline heart rate (beats/min), mean ± SD	75.9 (13.6)	70.4 (11.9)	0.154	1.05 (0.98–1.11)
Total propofol dose (mg), mean ± SD	264.9 (147.4)	233.5 (138.3)	0.339	1.00 (1.00–1.01)
Total fentanil dose (μg), mean ± SD	141.8 (96.0)	112.5 (53.4)	0.17	1.01 (1.00–1.02)
Procedure time (min), mean ± SD	75.2 (28.5)	61.5 (29.3)	0.112	1.02 (1.00–1.05)

Qualitative data are presented as *n* (%). The STOP-Bang questionnaire consists of eight dichotomous (Yes/No) questions: (1) snoring, (2) daytime tiredness, (3) observed apnea, (4) high blood pressure, (5) body mass index ≥ 30, (6) age ≥ 50 years, (7) neck circumference ≥ 40 cm, and (8) male sex. The total score ranges from 0 to 8.

*ASA* American Society of Anesthesiologists, *CI* confidence interval, *COPD* chronic obstructive pulmonary disease, *CXR* chest X-ray, *DBE* double-balloon enteroscopy, *ERCP* endoscopic retrograde cholangiopancreatography, *ESD* endoscopic submucosal dissection, *EUS-FNA* endoscopic ultrasound-guided fine-needle aspiration, *OR* odds ratio, *SD* standard deviation, *SpO*_*2*_ peripheral capillary oxygen saturation.

### Respiratory abnormality detectability

During treatment, oxygen desaturation of SpO_2_ < 90% occurred in 12 patients. Respiratory abnormalities were detected in ≥ 50% of the desaturation events in 9 (75.0%) patients with capnography and 11 (91.7%) patients with nasal pressure monitoring (*p* = 0.59, odds ratio (OR) 0.29; 95% CI 0.004–4.31). Respiratory abnormality detectability was acceptably high for both respiratory monitoring systems and did not differ between the monitors.

### False alarm

Of the 43 patients who underwent endoscopic treatment, false alarms were detected in 7 (16.3%) patients with capnography and 11 (25.6%) patients with nasal pressure monitoring (*p* = 0.427, OR 0.57; 95% CI 0.17–1.84). False alarms were often detected more than once in the same patient: 11 times with capnography versus 19 times with nasal pressure monitoring. Details of the false alarms are shown in Table 4. Although the nasal pressure monitor had a higher false alarm rate during treatment than capnography, no significant differences were observed.

**Table 4:** Number of false alarms.

Number of false alarms	Capnography		Nasal pressure monitoring	
	Number of cases	Ratio (95% CI)	Number of cases	Ratio (95% CI)
0	36	83.7% (69.3–93.2)	32	74.4% (58.8–86.5)
1	5	11.6% (3.9–25.1)	6	14.0% (5.3–27.9)
2	1	2.3% (0.6–12.3)	3	7.0% (1.5–19.1)
3	0	0% (0–8.2)	1	2.3% (0.6–12.3)
4	1	2.3% (0.6–12.3)	1	2.3% (0.6–12.3)
*p*	0.75			

The *p*-value was calculated using Fisher’s exact test.

*CI* confidence interval.

### Respiratory abnormalities detected by each monitor

Table 5 shows the respiratory abnormalities that each monitor could detect when SpO_2_ decreased to < 90%. The total number of hypoxemic events differed between nasal pressure monitoring and capnography. However, this discrepancy is attributed to a lack of some data. Obstructive respiratory disorder was detected only by nasal pressure monitoring. However, no significant differences were observed.

**Table 5:** Respiratory abnormalities when SpO_2_ decreases to < 90%.

Respiratory abnormalities	Capnography	Nasal pressure monitoring	*p*	OR (95% CI)
Apnea > 20 s	8/22 (36.4%)	9/23 (39.1%)	1	0.89 (0.22–3.50)
Hypopnea	6/22 (27.3%)	5/23 (21.7%)	0.738	1.34 (0.28–6.74)
Respiratory rate < 8	2/22 (9.1%)	3/23 (13.0%)	1	0.67 (0.05–6.55)
Obstructive disorder	0/22 (0%)	5/23 (21.7%)	0.049	0.00 (0.00–1.04)

Values are presented as the number of events/*n* (%). Multiple respiratory abnormalities were sometimes detected in one event.

*CI* confidence interval, *OR* odds ratio, *SpO*_*2*_ peripheral capillary oxygen saturation.

### Representative cases

**Figure 2:**
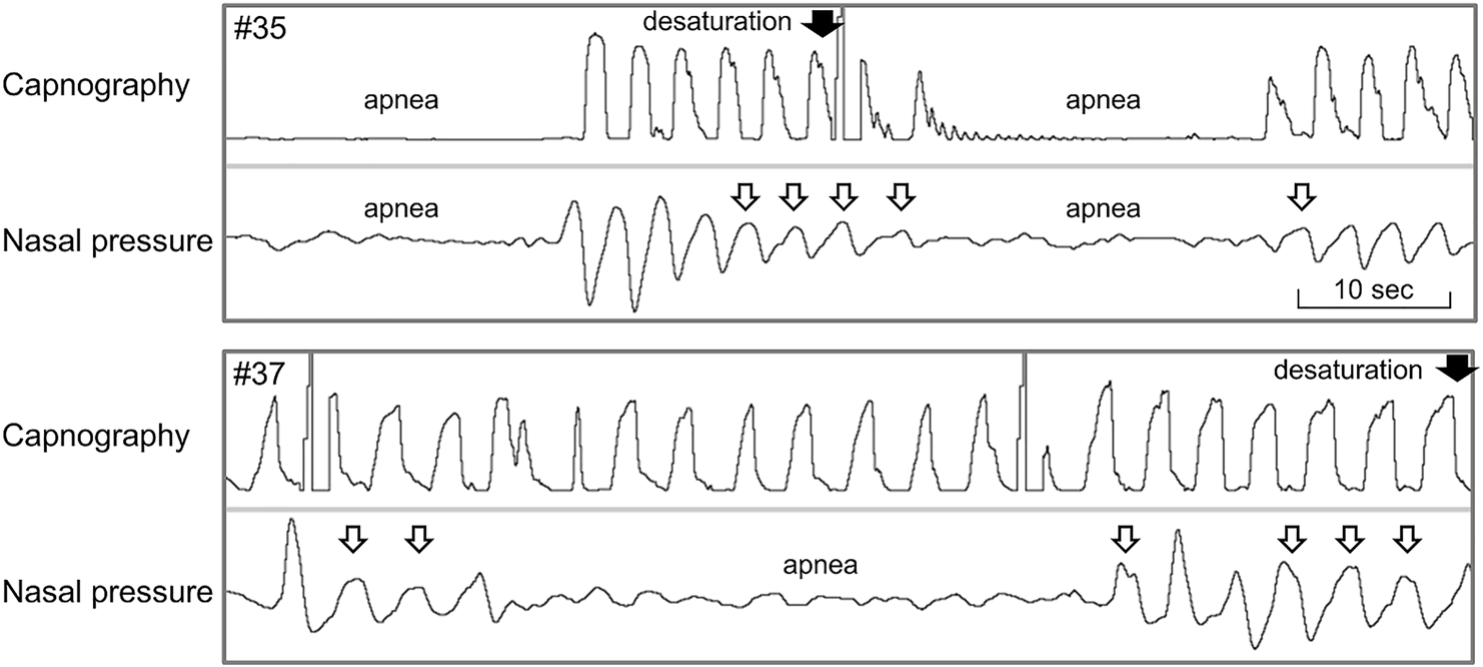
Waveform examples. Top, capnography; bottom, nasal pressure monitoring. Arrows (black) indicate the point of SpO_2_ < 90% in Patient #35 and SpO_2_ < 95% in Patient #37. Arrows (white) indicate obstructive respiratory disorder, revealing a flattened waveform pattern. No desaturation occurred after the second short period of apnea in patients #35.

Figure 2 shows representative oxygen desaturation waveforms. In Patient #35, apnea > 20 s was observed with the nasal pressure monitor and capnography before the first oxygen desaturation of SpO_2_ < 90%. SpO_2_ recovered to 96% and then decreased to 92% due to apnea with capnography. However, nasal pressure monitoring detected hypopnea and obstructive respiratory disorder before apnea occurred. In Patient #37, the nasal pressure monitoring detected hypopnea and obstructive respiratory disorder when oxygen desaturation of SpO_2_ < 95% occurred. However, no respiratory abnormality was observed with capnography.

## Discussion

In this phase II study, no major safety issues were observed with the nasal pressure monitor as a novel approach to monitoring during invasive endoscopic procedures under sedation with propofol and fentanyl. SpO_2_ reduction below 90% occurred in 27.9% of patients managed with the nasal pressure monitor in addition to standard monitors such as pulse oximetry. Despite the additional use of opioids to propofol in this study, the hypoxemic incidence was comparable to that in previous reports (12–50%) under propofol-based sedation.

Of the 45 patients, two cases dropped out: one due to the cancellation of the endoscopic procedure itself and the other due to the non-display of the monitor due to the occlusion of the cannula by the patient’s body, causing inhibition of pressure transmission. No similar event had occurred since then by taking care not to press the cannula. Two adverse events clearly related to endoscopic procedures were observed, but they had no effect on the primary goal of this phase II study, that is, confirming the safety of nasal pressure monitoring. Furthermore, waveform analysis revealed that the nasal pressure monitoring system could detect respiratory abnormalities when oxygen desaturation episodes occurred. Therefore, nasal pressure monitoring can be used as a respiratory monitor during the endoscopic procedure under sedation to detect respiratory abnormalities before SpO_2_ drops. This study may be meaningful in that both the capnography sensor and the intranasal pressure sensor were attached to the same patients during the same procedure. During treatment, only the nasal pressure monitor waveform could be referenced. Therefore, it may not be possible to compare the two monitors fairly. However, we could compare the waveforms of the two monitors with the same timing. Therefore, the characteristics of each monitor could be known. As shown in the representative examples, combining the information from both monitors might lead to a better understanding.

According to waveform analysis, there were more patients with high detectability of respiratory abnormalities at oxygen desaturation by the nasal pressure monitor than by capnography. However, no significant differences were observed. As previously reported, even if capnography did not display respiratory abnormalities, hypoxic events occurred in this study. The nasal pressure monitor also could not detect respiratory abnormalities before all hypoxic events^15,29–31^. Regarding false alarms, which were uniquely defined in this study, there were more false alarms with the nasal pressure monitor than with capnography. However, no significant differences were observed between the two monitors in this population. One possibility is that nasal airway obstruction was caused by endoscopy, which impeded nasal breathing and made oral breathing in which there was insufficient intranasal pressure.

This study also thoroughly examined respiratory abnormalities during oxygen deficiency, demonstrating that only the nasal pressure monitor could detect obstructive disorders, which capnography did not (Table 5). However, no significant differences in the respiratory abnormalities detected by the two monitors were observed. These results need to be verified with a phase III study using a larger population to reveal differences in features between the two monitors.

In this study, ASA class I and allergies were risk factors associated with a decrease in SpO_2_ < 90%, although ASA class > II was reported to be a risk factor for developing sedation-associated complications^32^. Confounding factors should have an impact, but a multivariate analysis was not performed due to the small population size. Because determining the risk factors for SpO_2_ decrease was not the primary goal of this study, a larger sample size is needed to evaluate the risk factors.

This study has several limitations. First, this was a single-center study conducted at Chiba University. Second, waveform analysis may be biased due to physician subjectivity, even though waveform analysis was performed by three physicians, one of whom was an expert in waveform analysis, and each respiratory abnormality was defined in detail. Third, a waveform analyst examined respiratory abnormalities at the point of oxygen desaturation a few weeks after the procedure to compare the two monitoring systems, even though it is critical that the physician in charge of sedation be able to make a decision regarding respiratory abnormalities during the procedure. This point should be investigated further in future research. Fourth, since the trial was planned as a phase II pilot trial to evaluate the safety and feasibility of nasal pressure monitoring compared to capnography, sample size and event size were low even though the statistically demanded size was reached. Therefore, the study was underpowered to find differences in hypoxic event rates and false alarms for nasal pressure monitoring and capnography. Fifth, since the Japanese guidelines for sedation in gastroenterological endoscopy describe the effectiveness of the combined use of propofol and fentanyl for ERCP^33^, we used both drugs for sedation in this study. The sedation with propofol alone adopted in many other countries might lead to different results. Furthermore, apnea > 3 min without oxygen desaturation was used as the definition of false alarm events. However, literature described shorter durations of apneas without desaturation, as presented in Fig. 2. We did not include these apneas in the false alarm events because the detection of the non-hypoxemic apneas is expected for both respiratory monitoring systems and is not an error. However, the 3-min definition used in this study for the false alarm event might have been too long to capture many falsely detected events lasting for 1–3 min by both monitoring systems.

In conclusion, this study confirmed the safety of the nasal pressure monitor to proceed to a phase III trial. This monitoring method during an endoscopic procedure that requires sedation may be useful in detecting respiratory abnormalities before SpO_2_ drops.

## Supplementary Information

Supplementary Information 2.

Supplementary Information 1.

## Data Availability

All data generated or analysed during this study are included in this published article (and its Supplementary Information files).
