# Supplementary material for: Phase II study comparing nasal pressure monitoring with capnography during invasive endoscopic procedures: a single-center, single-arm trial
Source: Sci Rep. 2023 Jan 23;13:1265. doi: 10.1038/s41598-023-28213-y (PMC9871023; doi:10.1038/s41598-023-28213-y)
Supplement: Supplementary file 1 — Supplementary Information 2. [file 41598_2023_28213_MOESM1_ESM.docx]

**Study Protocol**

**Phase II and phase III study regarding the Safety and Usefulness of a Nasal Pressure Monitor in Invasive Endoscopy Under Sedation**

**Principal investigator**

Prof. Naoya Kato, Department of Gastroenterology, Chiba University Hospital

**Research Administrator**

Prof. Shiro Isono, Department of Anesthesiology, Pain and Palliative Care Medicine, Chiba University Hospital

Correspondence to: 1-8-1 Inohana, Chuo-ku, Chiba-shi, Chiba 260-8677, JAPAN
Tel: +81 43-222-7171

Revision History

| Creation Date | Version |
| --- | --- |
| July 31, 2019 | Version 1.0 **(first version)** created |
| August 8, 2019 | Version 1.1 (revised) |
| September 3, 2019 | Version 1.2 (revised) |

**List of abbreviations**

| ERCP | Endoscopic retrograde cholangiopancreatography |
| --- | --- |
| ESD | Endoscopic Submucosal Dissection |
| EUS-FNA | Endoscopic UltraSound-guided Fine Needle Aspiration |
| DBE | Double balloon enteroscope |
| ASA | American Society of Anesthesiologists |
| BIS | Bispectral Index |
| BMI | Body Mass Index |
| CRF | Case Report Form |
| CTCAE | Common terminology criteria for adverse events |
| ECOG-PS | Eastern Cooperative Oncology Group  Performance status |
| ETCO2 | End tidal CO2 |
| FAS | Full analysis set |
| FRS | Face rating scale |
| IDMC | Independent Data Monitoring Committee |
| JCOG | Japan Clinical Oncology Group |
| NYHA | New York heart association |
| PMDA | Pharmaceuticals and Medical Devices Agency |
| PPS | Per protocol set |
| SAF | Safety analysis set |
| SAS | Sleep Apnea Syndrome |
| VAS | Visual analog scale |

**Index**

[0. Study Outline 6](#_Toc4508435)

[1. Introduction 9](#_Toc4508436)

[2. The Objectives and Necessity of Research 9](#_Toc4508437)

[3. Eligible Participants 9](#_Toc4508438)

[3.1. Selection Criteria 9](#_Toc4508439)

[3.2. ExclusionCriteria 10](#_Toc4508440)

[4. Consent of Participants 10](#_Toc4508441)

[4.1. Preparation and Revision of Consent Form and other Explanatory Documents 10](#_Toc4508442)

[4.2. Timing and Method of Obtaining Consent 10](#_Toc4508443)

[4.3. Information for Study Participants 11](#_Toc4508444)

[5. Study Method 13](#_Toc4508445)

[5.1. Study Design 13](#_Toc4508446)

[5.2. Study Outline 13](#_Toc4508447)

[5.3. Target Number of Participants and Study Period 13](#_Toc4508448)

[5.4. Institution Registration and Case Registration/Assignment Method 1](#_Toc4508449)3

[5.4.1. Case Registration/Assignment Method 14](#_Toc4508450)

[5.4.2. Assignment Adjustment Factors 14](#_Toc4508451)

[5.4.3. Registration Center Location 14](#_Toc4508452)

[5.5. Handling of Ineligible Patients 14](#_Toc4508453)

[5.6. Treatment Schedule and Method 15](#_Toc4508454)

[5.7. Response to Respiratory Abnormalities 16](#_Toc4508455)

[5.8. Standards for Discontinuation in Individual Cases 16](#_Toc4508456)

[5.9. After the Completion of the Study 16](#_Toc4508457)

[6. Testing Equipment 17](#_Toc4508458)

[6.1. Testing Equipment Overview 17](#_Toc4508459)

[6.2. Storage of Testing Equipment 17](#_Toc4508460)

[6.3. Important Findings regarding the Testing Equipment 17](#_Toc4508460)

[6.3.1. Capnography (ETCO2) Sensor and Nasal Adaptor 17](#_Toc4508465)

[6.3.2. Nasal Sensor Cable and Resp-NP Cannula 17](#_Toc4508466)

[6.3.3. Monitor 17](#_Toc4508467)

[7. Examination/Testing/Evaluation Items, Methods and Schedule 19](#_Toc4508461)

[7.1. Schedule and Procedure 19](#_Toc4508462)

[7.1.1. Screening 20](#_Toc4508463)

[7.1.2. Examination, Testing and Evaluation 21](#_Toc4508464)

[7.1.2.1.During Treatment. 21](#_Toc4508464)

[7.1.2.2.At the End of Treatment 21](#_Toc4508464)

[7.1.2.3.After Procedure (30 min, 1 hr, 2 hrs and 3 hrs). 22](#_Toc4508464)

[7.1.2.4.Following Day 22](#_Toc4508464)

[8. Handling Adverse Event 22](#_Toc4508469)

[8.1. Definition of Adverse Event 22](#_Toc4508470)

[8.2. Definition of Disease, etc 22](#_Toc4508471)

[8.3. Dealing with Participants when Adverse Events Occur 23](#_Toc4508472)

[8.4. Obtaining Information regarding Adverse Events 23](#_Toc4508473)

[8.5. Adverse Event Reporting Period 23](#_Toc4508474)

[8.6. Reporting Details Required for Evaluating Adverse Events 23](#_Toc4508475)

[8.6.1. Adverse Event Recoverability and Causal Relationship with Testing Equipment 23](#_Toc4508476)

[8.6.2. Adverse Event Predictability Evaluation 24](#_Toc4508477)

[8.7. Expected Adverse Events 24](#_Toc4508478)

[8.7.1. Expected Adverse Events Associated with ERCP, EUSFNA, ESD and DBE 24](#_Toc4508479)

[8.7.2. Adverse Events due to Propofol 24](#_Toc4508480)

[8.7.3. Adverse Events due to Fentanyl 24](#_Toc4508481)

[9. Handling Serious Adverse Events 24](#_Toc4508482)

[9.1. Definition of Serious Adverse Event 24](#_Toc4508483)

[9.2. Serious Adverse Events Subject to Reporting 24](#_Toc4508484)

[9.3. Reporting Disease, etc., and Defects 25](#_Toc4508485)

[9.4. Serious Adverse Event (Disease, etc./Defect) Reporting Procedure 26](#_Toc4508486)

[9.5. Regural Reports 2](#_Toc4508487)6

[10. Evaluation Items 27](#_Toc4508488)

[10.1. Primary Endpoint 27](#_Toc4508489)

[10.2. Secondary Endpoints 27](#_Toc4508490)

[10.3. Exploratory Endpoint 27](#_Toc4508491)

[11. Statistical Considerations 2](#_Toc4508492)8

[11.1. Analysis Population 28](#_Toc4508493)

[11.1.1. Full Analysis Set (FAS) 28](#_Toc4508494)

[11.1.2. Per Protocol Set (PPS) 28](#_Toc4508495)

[11.1.3. Safety analysis se (SAS) 28](#_Toc4508495)

[11.2. Target Number of Cases and Basis for Selection 28](#_Toc4508496)

[11.3. Handling Case Records 29](#_Toc4508497)

[11.4. Handling Data 29](#_Toc4508498)

[11.5. Statistical Analysis Items and Plan 29](#_Toc4508499)

[11.5.1. Patient Background Analysis 29](#_Toc4508500)

[11.5.2. Usefulness Analysis 29](#_Toc4508501)

[11.5.2.1.Primary Analysis 29](#_Toc4508501)

[11.5.2.2.Secondary Analysis 30](#_Toc4508501)

[11.5.3. Safety Analysis 3](#_Toc4508504)**0**

[11.5.4. Intermediate Analysis 3](#_Toc4508505)**0**

[11.6. Independent Data Monitoring Committee (IDMC) 30](#_Toc4508506)

[11.7. Final Analysis 3](#_Toc4508507)0

[12. Compliance with Study Protocol and Nonconformity Management 31](#_Toc4508508)

[13. Changes regarding Study Protocol, Case Report or Analysis Plan 31](#_Toc4508509)

[13.1. Revising Study Protocol and Case Report 31](#_Toc4508510)

[13.2. Revising Analysis Plan 31](#_Toc4508511)

[14. Cancellation, Discontinuation or Completion of the Study 32](#_Toc4508512)

[14.1. Standard for Canceling or Discontinuing the Study 32](#_Toc4508513)

[14.2. Procedure to Cancel or Discontinue the Study 32](#_Toc4508514)

[14.3. Procedure for Cancelling or Discontinuing the Study at the Individual Institution 32](#_Toc4508515)

[14.4. Completion of the Study 32](#_Toc4508516)

[15. Data Management 33](#_Toc4508517)

[15.1. Data Entry Method and Management 33](#_Toc4508518)

[15.2. Identification of First-hand Entry into CRFs (Raw Data) 33](#_Toc4508519)

[16. Storing Raw Data and Other Records 33](#_Toc4508520)

[17. Direct Perusal of Raw Data 34](#_Toc4508521)

[18. Quality Control and Assurance in the Study 34](#_Toc4508522)

[18.1. Quality Control 34](#_Toc4508523)

[18.2. Quality Assurance 34](#_Toc4508524)

[19. Ethics 34](#_Toc4508525)

[20. Confidentiality 34](#_Toc4508526)

[21. Approved Clinical Study Review Board 3](#_Toc4508527)4

[22. Expected Advantages/Disadvantages Arising from the Study 3](#_Toc4508528)5

[22.1. Expected Advantages 3](#_Toc4508529)5

[22.2. Expected Disadvantages 35](#_Toc4508530)

[23. Compensation for Health Damage and Insurance 35](#_Toc4508531)

[23.1. Compensation for Health Damage 35](#_Toc4508532)

[23.2. Enrollment in Clinical Trial Insurance (Compensation Insurance) 35](#_Toc4508533)

[23.3. Enrollment in Liability Insurance 36](#_Toc4508534)

[24. Monetary Payments 36](#_Toc4508535)

[25. Conflicts of Interest and Research Funds 36](#_Toc4508536)

[26. Disclosure of Information regarding the Study 36](#_Toc4508537)

[27. Announcement of Results 36](#_Toc4508538)

[27.1. Annoucement Method 36](#_Toc4508539)

[27.2. Rules for Announcement 37](#_Toc4508540)

[28. Framework of Study 37](#_Toc4508541)

[29. References 38](#_Toc4508542)

1. **Study Outline**

| **Title** | **Phase II and phase III study regarding the Safety and Usefulness of a Nasal Pressure Monitor in Invasive Endoscopy Under Sedation** |
| --- | --- |
| **Objective** | To conduct a safety evaluation of a novel nasal pressure monitor used for patients who undergo invasive endoscopy (endoscopic retrograde cholangiopancreatography (ERCP), endoscopic submucosal dissection (ESD), endoscopic ultrasound guided fine needle aspiration (EUS-FNA), and oral double-balloon enteroscopy (DBE)), and evaluate its usefulness by means of a randomized single-blind controlled study to show its non-inferiority compared with capnography, which is the current standard of monitoring respiration. |
| **Study Design** | Safety Evaluation (Phase II): Single-center, single-arm study  Usefulness Evaluation (Phase III): Single-center, randomized, single-blind controlled study. |
| **Equipment** | Nasal pressure monitor |
| **Evaluation Items** | Nasal pressure monitor and capnography CO_2_ sensor (TG-920P) |
| **Selection Criteria** | Patients who satisfy all of the following conditions shall be the subjects of the study.  Patients who undergo invasive endoscopic treatment (ERCP, ESD, EUS-FNA and DBE) under sedation at the Endoscopy Center of Chiba University Hospital and who meet the following criteria, but not the exclusion criteria.   1. Aged 20 years or older (regardless of sex) 2. Patients who receive a thorough explanation regarding participating in the study prior to the commencement of the study, and who provide their written consent. 3. Patients with SpO2 of ≥95% (room air). 4. Patients with systolic pressure of ≥90 mmHg. |
| **Exclusion Criteria** | Patients who correspond to any of the following conditions shall be excluded from the study.   1. Patients who regularly take an oral opioid formulation or oral benzodiazepine (with the exception of cases where it is taken internally as a sleep inducer before going to bed). 2. Patients who register with another clinical study within four weeks of registering for this study. 3. Patients with a drug abuse problem or whose physical, psychological or social circumstances may hinder their participation in the study or interfere with the evaluation of the results of the study. 4. Patients with the following cardiovascular diseases: 5. Patients with NYHA Class 3 or above heart failure (see Reference). 6. Patients with a coronary disorder with current symptoms or a history of myocardial infarction during the 24 weeks prior to registering with the study. 7. Patients with arrhythmia that requires control using antiarrhythmic drugs, such as beta blockers or digoxin (CTCAE Version 4.0 Japanese translation Grade 3 or higher). 8. Patients with poorly controlled hypertension. 9. Patients classified as Class ≥4 under the American Society of Anesthesiologists (ASA) Physical Status Classification System (see Reference). 10. Patients with bradycardia (heart rate <50). 11. Patients with a serious thyroid disorder. 12. Patients who are or may be pregnant or who are trying to become pregnant, or who are breast-feeding. (A pregnancy test must be performed whenever pregnancy is suspected.) 13. Patients who may potentially have an allergic reaction to propofol and/or fentanyl. 14. Other cases where it is deemed that the study may harm the safety of the patient or that it is too difficult for the patient to comply with the study protocol. |
| **Evaluation Items** | **Primary Endpoint**  Hypoxemia occurrence rate (SpO2<90%): number of cases / total number of eligible participants  **Secondary Endpoints**  Frequency of apnea (20 seconds or more)  Degree of participant satisfaction (VAS)  Intraoperative coordination (VAS)  Frequency of adverse events  **Exploratory Endpoints**  Comparison with capnography waveform  Waveform analysis at the time of respiratory abnormality  Respiratory abnormality-related factors (includes obesity, age, history of smoking, drug administration dose, treatment duration and SAS). |
| **Study Method** | A single-arm study to evaluate the safety of the nasal pressure monitor will be carried out with patients who undergo invasive endoscopy, followed by a randomized comparative study to validate its usefulness. In the randomized comparative study, patients will be single-blinded and divided into two groups: a nasal pressure monitor group and a capnography group. Sedatives will be administered to each group based exclusively on data from their respective monitors.  Sedation of both groups will be carried out using propofol and fentanyl. The sedation depth in each group will be kept between 60 and 80 on the bispectral index (BIS) using the respective monitor. Monitoring will continue until three hours after the completion of the endoscopic procedure. |
| **Target No. of Patients** | Safety Evaluation (Phase II): 45  Usefulness Evaluation (Phase III): 224 (112 in each group) |
| **Study Period** | ● Overall duration: 24 months (from the initial announcement on the Japan Registry for Clinical Trials (jRCT) until August 31, 2021)  * Detailed Schedule  Safety Evaluation (Phase II): 10 months (from the initial announcement on the jRCT until June 30, 2020)  Usefulness Evaluation (Phase III): 20 months (from January 1, 2020 until August 31, 2021)  ● Registration period: 18 months (from the initial announcement on the jRCT until February 28, 2021)  * Registration Details  Registration for Safety Evaluation (Phase II): 4 months (from the initial announcement on the jRCT until December 31, 2019)  Registration for Usefulness Evaluation (Phase III): 14 months (from January 1, 2020 until February 28, 2021) |
| **Number of Participating Institutions** | One |
| **Ethics Policy** | This clinical study will be carried out in compliance with ethical principles and clinical study methods based on the Declaration of Helsinki and other related regulatory requirements. |
| **Authorized Clinical Study**  **Review Board** | Prior to the commencement of this clinical study, the Authorized Clinical Study Review Board of the participating institution will examine the ethical, scientific and medical appropriateness of of the study. The study will commence after approval has been received from the Authorized Clinical Study Review Board. The Authorized Clinical Study Review Board will continue to review the performance of the study at least once a year. |

1. **Introduction**

In many cases that require highly invasive endoscopic procedures, such as endoscopic retrograde cholangiopancreatography (ERCP) and endoscopic submucosal dissection (ESD), sedatives are added to intravenous anesthesia in order to alleviate the pain experienced by patients. As there is the risk of hypoxemia during and following endoscopy under sedation due to the suppressive effect of the sedative on the respiratory system, use of an appropriate respiration monitor (monitored anesthesia care: MAC) is recommended.[1.2] In terms of the monitoring equipment used in such situations, a pulse oximeter is an essential item, but in recent years capnography, which measures end-tidal CO2, is becoming popular and its usefulness is also being reported in meta-analysis.[3] However, problems associated with capnography have been pointed out, including declined reliability in endoscopic procedures that use CO2, data fluctuations associated with hiccupping, and false alarms triggered by displaced sensors. It has also been reported that on analyzing detailed waveform analysis obtained using capnography, in approximately half of the cases in which a drop in SpO2 was observed, it was not possible to identify the cause of the respiratory abnormality.[4]

Nasal pressure monitoring is used as a means of ascertaining respiration during sleep, which is similar to respiration under sedation, and the American Academy of Sleep Medicine (AASM) recommends the use of nasal pressure monitoring instead of capnography in the sleep apnea test [5].

Existing studies where nasal pressure monitoring was used to ascertain the respiratory status of patients under sedation include one in which nasal pressure monitoring was used during oral surgery and was able to detect respiratory abnormalities that were unable to be detected with a pulse oximeter,[6] and another report which stated that nasal pressure monitoring during ESD under sedation was able to detect respiratory abnormalities accompanied by a decline in oxygen levels on average 107 seconds faster than a pulse oximeter.[7]

The nasal pressure monitor that we use in this particular study is a novel respiration monitor based on nasal pressure transducers used for the sleep apnea test that was jointly developed by the Department of Anesthesiology, Pain and Palliative Care Medicine of Chiba University Hospital and Nihon Kohden Corporation.

Nasal pressure monitoring has the following merits over capnography: 1) It is not affected by carbon dioxide supply, 2) it reflects the semiquantitative change in single ventilation volume, and 3) changes in waveforms help differentiate obstructive and central respiratory abnormalities. The ability to distinguish between central respiratory abnormalities due to excessive drug administration and pharyngeal airway obstruction that requires airway management on the basis of waveform patterns makes it possible to take appropriate measures earlier and prevent the development of severe hypoxemia.

1. **The Objectives and Necessity of Research**

The objectives of this study are to confirm the safety of patient respiration status monitoring using a nasal pressure monitor that is an unapproved medical device still currently under development for use with patients undergoing invasive endoscopy (ERCP, ESD, EUS-FNA and DBE), and to evaluate its usefulness by comparing with capnography. As mentioned above, although capnography is currently a popular option for use in clinical practice, a number of problems have been pointed out. If we can therefore show the usefulness of the new respiration monitor, which is expected to be able to detect respiratory abnormalities and their cause prior to the development of hypoxemia, this will have a significant impact on clinical practice.

1. **Eligible Participants**
   1. **Selection Criteria**

Patients who undergo invasive endoscopic treatment (ERCP, ESD, EUS-FNA or DBE) under sedation at the Endoscopy Center of Chiba University Hospital and who meet the following criteria, but not the exclusion criteria.

1. Aged 20 years or older (regardless of sex)
2. Patients who receive a thorough explanation regarding participating in the study prior to the commencement of the study, and who provide their written consent.
3. Patients with SpO2 of ≥95% (room air).
4. Patients with systolic pressure of ≥90 mmHg.
   1. **Exclusion Criteria**
5. Patients who regularly take an oral opioid formulation or oral benzodiazepine (with the exception of cases where it is taken internally as a sleep inducer before going to bed).
6. Patients who register with another clinical study within four weeks of registering for this study.
7. Patients with a drug abuse problem or whose physical, psychological or social circumstances may hinder their participation in the study or interfere with the evaluation of the results of the study.
8. Patients with the following cardiovascular diseases:
9. Patients with NYHA Class 3 or above heart failure (see Reference).
10. Patients with a coronary disorder with current symptoms or a history of myocardial infarction during the 24 weeks prior to registering with the study.
11. Patients with arrhythmia that requires control using antiarrhythmic drugs, such as beta blockers or digoxin (CTCAE Version 4.0 Japanese translation Grade 3 or higher).
12. Patients with poorly controlled hypertension.
13. Patients with bradycardia (heart rate <50).
14. Patients classified as Class ≥4 under the American Society of Anesthesiologists (ASA) Physical Status Classification System (see Reference).
15. Patients with a serious thyroid disorder.
16. Patients who are or may be pregnant or who are trying to become pregnant, or who are breast-feeding. (A pregnancy test must be performed whenever pregnancy is suspected.)
17. Patients who may potentially have an allergic reaction to propofol and/or fentanyl (allergic to eggs, soybeans, or coconuts).
18. Other cases where it is deemed that the study may harm the safety of the patient or that it is too difficult for the patients to comply with the study protocol.
19. **Consent of Participants**
    1. **Preparation and Revision of Consent Form and other Explanatory Documents**

The principal investigator or assistant investigators will prepare the consent form that is used to obtain the consent of the study participants to participate in the study and other explanatory documents using language that is as plain as possible. Such documents will be revised as deemed necessary.

The principal investigator will submit the prepared or revised consent form and explanatory documents to the Certified Clinical Study Review Board to obtain their approval.

- 1. **Timing and Method of Obtaining Consent**

1. **Obtaining Consent**

The principal investigator or assistant investigators will provide the consent form and other explanatory documents to participants and give a thorough explanation regarding “4.3 Information for Study Participants.” After confirming that the participants have thoroughly understood the details regarding the study, we will obtain their voluntary written consent prior to the commencement of the study (prior to the screening test).

1. **Procedures for Filling in the Consent Form**

When obtaining the consent of participants, the principal investigator or assistant investigators will explain details regarding the study to each participant, and enter the date of explanation, the name of the person who provided the explanation, and sign and/or seal the document. The participants will sign the consent form, entering the date of the day. After the consent form is signed, participants will be issued with copies of the consent form and the explanatory documents.

1. **Revising Explanatory Documents**

In cases where the principal investigator or assistant investigators revise the consent form or other explanatory documents based on new information that may affect the will of participants to consent, they will provide a further explanation to participants using the revised form and explanatory documents, and obtain consent in writing to continue with the study.

- 1. **Information for Study Participants**

The principal investigator will include the following details in the explanatory documents. The language used will be as plain as possible in order to enable participants to easily understand, and shall as far as possible contain the following matters.

1) The fact that the study involves research.

2) The objectives of the study.

3) The methods to be used in the study (the trial aspects of the study, the selection criteria for research participants, and the rate of assignment to each group.)

4) The scheduled period of participation in the study.

5) The scheduled number of participants in the study.

6) The expected clinical benefit and risks involved.

7) Whether or not there is another possible method of treatment for the participants, and the expected important benefits and risks associated with such treatment.

8) The compensation and treatment available for participants should they suffer any damage to their health in relation to the clinical study.

9) The fact that the participants will participate in the clinical study at their own discretion, and that they may refuse to participate or withdraw for the study at any time, and that they will not be disadvantaged by refusing to participate or by withdrawing from the study, nor will they lose the benefits that they are entitled to if they choose to no longer participate.

10) When information that may affect the decisions of participants concerning their participation in or their continuation with the study becomes available, such information will immediately be passed on to participants.

11) The conditions or reasons for ceasing to participate in the clinical study.

12) The fact that monitors, auditors, members of the Certified Clinical Study Review Board, and regulatory authorities may peruse the source documents concerning the medical treatment, while ensuring the confidentiality of the participants. The signing of the consent form constitutes the participant’s agreement to such perusal.

13) The fact that in cases where the results of the clinical research are released to the public, the confidentiality of participants will be maintained.

14) Details regarding payments, if there are times when the participants must bear some costs.

15) Details regarding payments, if there is a time when cash or something similar is provided to the participants (agreement concerning payment amount, etc.)

16) The names, titles and contact details of the principal investigator and assistant investigators.

17) The point of contact for the institutions that the participants should contact when they wish to know about the research or the rights of participants, or when they want to inquire regarding health damage that occurred in relation to the clinical study.

18) Matters that participants should comply with.

19) Matters decided in the Certified Clinical Study Review Board, which conducts investigations concerning the appropriateness of clinical studies, other matters regarding the Certified Clinical Study Review Board, and the content of notifications to the Minister of Health, Labour and Welfare.

20) Matters regarding the storage and disposal of samples.

21) Matters regarding the release of information concerning the clinical study.

22) Matters regarding intellectual property.

23) Matters regarding conflicts of interest

1. **Study Method**
   1. **Study Design**

This clinical study will be divided into a safety evaluation (Phase II) and a usefulness evaluation (Phase III). The safety evaluation will be conducted by means of a single-arm study, while the usefulness evaluation will be conducted as a confirmatory parallel-group, randomized, single-blinded, controlled trial using standard treatment.

- 1. **Study Outline**

Primary Endpoint

- Hypoxemia occurrence rate (SpO2<90%): number of cases / total number of eligible participants

Secondary Endpoints

- Frequency of apnea (≥20 seconds)
- Degree of participant satisfaction (VAS)
- Intraoperative coordination (VAS)
- Adverse event occurrence rate

Exploratory Evaluation

- Comparison with capnography waveform
- Waveform analysis at the time of respiratory abnormality
- Consideration of respiratory abnormality-related risk factors (including obesity, age, history of smoking, drug administration dose, treatment duration and SAS).
  1. **Target Number of Participants and Study Period**

Safety Evaluation (Phase II): 45

Usefulness Evaluation (Phase III): 224 (112 in each group)

● Overall Duration: 24 months (from the initial announcement on the Japan Registry for Clinical Trials (jRCT) until August 31, 2021)

*Safety Evaluation (Phase II): 10 months (from the initial announcement on the jRCT until June 30, 2020)

Usefulness Evaluation (Phase III): 20 months (from January 1, 2020 until August 31, 2021)

● Registration Period: 18 months (from the initial announcement on the jRCT until February 28, 2021)

*Registration for Safety Evaluation (Phase II): 4 months (from the initial announcement on the jRCT until December 31, 2019)

Registration for Usefulness Evaluation (Phase III): 14 months (from January 1, 2020 until February 28, 2021)

- 1. **Institution Registration and Case Registration/Assignment Method**

Cases are registered centrally at the data center (Chiba University Hospital).

Case registration and assignment will be carried out in accordance with the following section.

- - 1. **Case Registration/Assignment Method**

● Safety Evaluation (Phase II)

1. Registration should take place, as a rule, within 14 days of obtaining consent.
2. The principal investigator or assistant investigators will obtain signed consent forms and confirm that participants satisfy the eligibility criteria, and that there is no conflict with the exclusion criteria. Registration forms should be filled in for participants who signed the consent form and sent it to the data center by fax or delivered it in person. The content of registration forms is verified at the data center, and eligibility assessment results are sent back by fax.
3. Patients who are registered once will not be removed from the database. When the same person is registered multiple times, the initial registration information (Registration No.) should always be used.

　　When incorrect or multiple registrations are found, the registration center should be notified at once.

● Usefulness Evaluation (Phase III)

1. Registration should take place, as a rule, within 14 days of obtaining consent.
2. The principal investigator or assistant investigators will obtain signed consent forms and confirm that participants satisfy the eligibility criteria, and that there is no conflict with the exclusion criteria. Registration forms should be filled in for participants who signed the consent form and sent it to the data center by fax or delivered it in person. The content of the registration forms will be verified at the data center to confirm eligibility. Necessary information for eligible cases will be entered into the system, and all cases will be randomly assigned to either the capnography group or the nasal pressure monitor group at a ratio of 1:1. At the data center, assignment results will be checked and sent to the principal investigator or assistant investigators by fax.
3. Patients who are registered once will not be removed from the database. When the same person is registered multiple times, the initial registration information (Registration No.) should always be used.

When incorrect or multiple registrations are found, the registration center should be notified at once.

* The principal investigator and assistant investigators must not commence the study until registration and assignment of all participants have been completed.

- - 1. **Assignment Adjustment Factors**

Assignment adjustment factors for the usefulness evaluation are procedure (EUS-FNA, ERCP, ESD and DBE), obesity (BMI ≥25, <25), and sex.

- - 1. **Registration Center Location**

Chiba University Hospital Date Center

Business hours: 09:00 – 17:00

Tel; 043-221-7171 (Ext.6593)

Fax: 043-226-2644

- 1. **Handling of Ineligible Patients**

● Safety Evaluation (Phase II)

Patients who are not registered due to ineligibility or other reasons will be counted as non-registered participants and not included as study subjects. Such patients will receive an explanation from the principal investigator or assistant investigators regarding whether or not it is possible to register for the clinical study.

● Usefulness Evaluation (Phase III)

Patients who were not assigned to a group at the time of registration due to ineligibility or other reasons will be counted as non-registered participants and not included as study subjects. Such patients will receive an explanation from the principal investigator or assistant investigators regarding whether or not it is possible to register for the clinical study.

- 1. **Treatment Schedule and Method**

● Safety Evaluation (Phase II)

| Before entering the treatment room for invasive endoscopy | A signed consent form will be obtained from each participant, their eligibility verified, and participants registered. Only registration for the Safety Evaluation (Phase II) will be carried out, not assignment. |
| --- | --- |
| After entering the treatment room for invasive endoscopy | For the Safety Evaluation (Phase II), patients will be fitted with both a capnography monitor and a nasal pressure monitor. After the nasal pressure monitor is fitted, do not turn on the capnography monitor, only check the display of the nasal pressure monitor, and administer sedatives. The capnography waveforms will be recorded and used later for waveform analysis along with those from the nasal pressure monitor.  Propofol and fentanyl will be used as sedatives, and will be administered by an endoscopist who has been fully trained in sedation by an anesthesiologist. The strength of the sedatives during the procedure will be controlled between BIS 60 and 80 as shown on the monitor in both groups. Prior to the administration of sedatives, oxygen will be administered through a transnasal catheter at a rate of 2 l/min. Local pharyngeal anesthesia (8% lidocaine spray) will be sufficiently administered until the vomiting reflex disappears. Propofol and fentanyl will be administered in accordance with a predetermined protocol (see below), while monitoring the patient’s sedation depth and suitably adjusting the dose of each drug. Administration will be stopped when the procedure has been completed, and if respiration rate drops to ≤8 bpm, naloxone will be administered. Patient response will be verified by calling out to the patient, and after checking that there are no cardiorespiratory dynamic problems, the patient will be allowed to leave the treatment room.  In cases where SpO_2_ is observed to have declined (<90), apnea lasts for ≥20 seconds, or a respiratory abnormality (≤8 bpm or abnormal waveform) accompanied by a decline in SpO_2_ (<95%) is observed during sedation, the doctor in change of sedation will report the respiratory abnormality to the endoscopy team, and measures will be taken to improve respiration (see 5.7). When such measures have been taken, details regarding the respiratory abnormality that caused them are recorded in the check sheet.  Monitoring data will be analyzed for up to three hours after the treatment in the Safety Evaluation (Phase II). Later, assistant investigators will analyze the monitor waveforms. |

● Usefulness Evaluation (Phase III)

| Before entering the treatment room for invasive endoscopy | Patients who undergo invasive endoscopy will be assigned to either the nasal pressure monitor group or the capnography group and single-blinded by going through the following steps.   1. A signed consent form will be obtained from each participant, their eligibility verified, and participants registered. 2. Assignment results will be sent from the registration center to the principal investigator or assistant investigators. |
| --- | --- |
| After entering the treatment room for invasive endoscopy | In the Usefulness Evaluation (Phase III), both the capnography group and the nasal pressure monitor group will be sedated using one type of monitor, which will be the one used in the group to which they were assigned.  The method of sedation and response used in cases of respiratory abnormality are the same as those used in the Safety Evaluation (Phase II) (see 5.6).  In the usefulness evaluation, monitoring data will be analyzed for up to one hour after treatment. Later, assistant investigators will analyze the monitor waveforms. |

- 1. **Response to Respiratory Abnormalities**

In cases where SpO_2_ is observed to have declined (<90), apnea lasts for ≥20 seconds or a respiratory abnormality (≤8 bpm or abnormal waveform) accompanied by a decline in SpO_2_ (<95%) is observed during sedation, the doctor in change of sedation will report the respiratory abnormality to the endoscopy team, and measures will be taken to improve respiration (see 5.7).

When SpO_2_ drops to below 90%, oxygen will be increased, the patient will be stimulated (call their name), airway management will be carried out (head tilt, chin lift, or postural change), and propofol and fentanyl administration will be stopped or adjusted as required. In cases where such procedures do not improve the situation, a nasal airway tube will be inserted or the endoscope will be removed and artificial ventilation provided using an Ambu bag (a bag valve mask).

If SpO_2_ is 90–94%, first increase the oxygen supply (maximum of 6 l). If SpO_2_ does not improve, if the cause can be assumed from the monitor waveforms, deal with the cause. That is to say, in cases of apnea, infrequent respiration or low respiration, stimulate the patient, reduce and/or stop propofol and fentanyl, and in cases of obstructive ventilatory impairment, maintain airway patency. In cases where apnea that lasts for ≥20 seconds is observed without a decline in SpO_2_, the doctor in charge of sedation will consider response measures, including observation.

- 1. **Standards for Discontinuation in Individual Cases**

In cases where the following criteria are met, the principal investigator or assistant investigators will discontinue the study. However, testing and evaluation will be continued according to the schedule.

1. When adverse events occur that make the continuation of the study difficult, and the principal investigator or assistant investigators decide it is necessary to discontinue the study.
2. When eligible participants request it to be discontinued.
3. In other cases where the principal investigator or assistant investigators decide that it is impossible for eligible participants to continue with the study.
4. When the Research Administrator decides to discontinue the study.

In cases that correspond to items other than 1) above, the date on which the study is discontinued shall not be the date on which the event occurred that caused such discontinuation, but the date on which the principal investigator or assistant investigator decides to discontinue the study.

- 1. **After the Completion of the Study**

Even after the completion of the study we will endeavor to make the best prevention, diagnosis and treatment methods obtained from the results of the study available to eligible participants of the study.

1. **Testing Equipment**
   1. **Testing Equipment Overview**

Testing equipment (novel nasal pressure monitor, general name: Multi-parameter Multi-function Monitor 33586003) has been developed by Nihon Kohden Corporation and the Department of Anesthesiology, Pain and Palliative Care Medicine of Chiba University Hospital based on their joint research. The nasal pressure monitoring sensor and the software for reading the sensor, which are connected to the patient monitor, are not approved as medical devices, but the patient monitor connected to them that displays the waveform has been approved (see the table below). The technology employed in this respiration monitoring system is the nasal pressure monitoring method that is actually used in nocturnal polysomnography. Changes in nasal pressure detected through a nasal cannula are measured with this equipment, and the fluctuations in pressure are converted to create respiratory waveforms for each patient.

- 1. **Storage of Testing Equipment**

The equipment to be tested (novel nasal pressure monitor), ETCO_2_ sensor and the nasal pressure sensor cable will be safely kept in accordance with the attached Testing Equipment Management Procedures.

- 1. **Important Findings regarding the Testing Equipment**
     1. **Capnography (ETCO_2_) Sensor and Nasal Adaptor**

There are no known reports of defects in ETCO_2_ sensors or adaptors that may cause adverse events. However, when using the equipment it is important to bear in mind the following:

• For patients with low ventilation volume, when the volume of respiratory dead space increases over ventilation volume, proper ventilation may not occur. In some cases, CO_2_ may enter the dead space and cause inaccurate measurements or make the detection of apnea difficult.

• When a patient is administered a volatile anesthetic drug during anesthesia, measurement errors may occur.

• For patients who are fitted with a nasal tube, take care when fitting the sensor not to put pressure on or dislodge the nasal tube.

* Reference: CO_2_ Sensor Kit TG-900 Series package insert.

- - 1. Nasal Sensor Cable and Resp-NP Cannula

As there is no existing summary document for the nasal pressure sensor cable, the package insert for a portable sleep apnea tester will be used for reference. Although the package insert mentions the possibility of an increase in temperature and pressure necrosis on the place where the SpO_2_ probe is attached (wrist), the SpO_2_ probe is not included among the equipment to be tested. There are no known reports of defects in Resp-NP cannulas that may cause adverse events.

* Reference: Portable Sleep Apnea Tester SAS-2100 package insert.

　　　　　　Respiratory Test Sensor Cannula YH Series package insert.

- - 1. **Monitor**

Please refer to the package insert for the CSM-1000 Series Bedside Monitor (including CSM-1901) that has been approved under the Pharmaceutical Affairs Law and which also displays SpO_2_. The software for reading the nasal pressure sensor is not approved under the Pharmaceutical Affairs Law.

* Reference: CSM-1000 Series Bedside Monitor package insert.


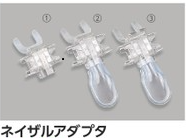

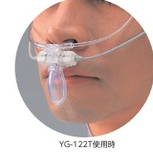


Figure1. Left is Capnography sensor, and Right is Nasal adaptor of Capnography Sensore.


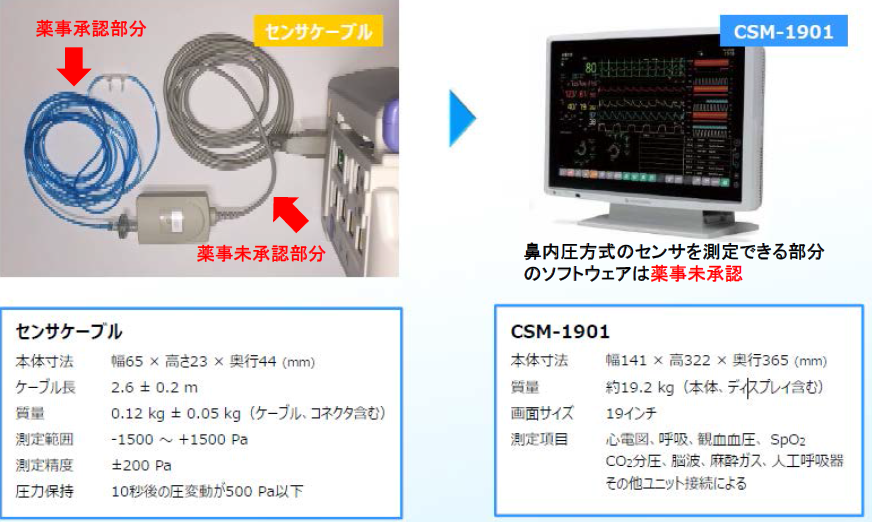


Figure2. Left is a nasal pressure monitor’s cannula and sensor. Right is the monitor to connect the Capnography and a nasal pressure sensor.

**Table 1. Overview of Testing Equipment Used in the Study**

| **General Name (Tentative Name for Unreleased Products)**  **Abbreviation, etc.** | **Model No. (Approval No.)** | **Unapproved** | **National Health Insurance Listing** | **Manufacturer** |
| --- | --- | --- | --- | --- |
| CO_2_ module 36552000 (capnography sensor) | CO_2_ sensor kit  (21400BZZ00536000) | No | No | Nihon Kohden  Corporation |
| CO_2_ module 36552000 *Attachment (ETCO_2_ nasal adaptor) | Nasal adaptor *Attachment for the CO_2_ sensor kit above. | No | No | Nihon Kohden  Corporation |
| Nasal pressure sensor cable | Resp-NP sensor | Yes | No | Nihon Kohden  Corporation |
| Resp-NP cannula | YH series respiratory test sensor cannula (14B2X10004TR0007). | No | Yes | Nissei Eco  Co., Ltd. |
| Multi-item monitor with major parameters 33586003 (monitor) | CSM-1000 Series Bedside Monitor Lifescope G *Including CSM-1901 (22500BZX00483000) | Partial  Unapproved | No | Nihon Kohden  Corporation |

1. **Examination/Testing/Evaluation Items, Methods and Schedule**
   1. **Schedule and Procedure**

The examination, testing and evaluation schedule will be as shown in the table below. The principal investigator or assistant investigators will carry out examination and testing, etc., in accordance with the schedule.

| Timing  Items | Screening  Test | During Treatment | End of  Treatment | After Treatment | | | | Following Day/  When Discontinued _g_ |
| --- | --- | --- | --- | --- | --- | --- | --- | --- |
|  |  |  |  | 30 min | 1 hr | 2 hrs | 3 hrs |  |
| Permissible range (days) | -7 | 0 |  |  |  |  |  |  |
| Consent obtained | ● |  |  |  |  |  |  |  |
| Patient background | ● |  |  |  |  |  |  |  |
| Subjective symptoms | ● | ● | ● | ● | ● | ● | ● | ● |
| Vital signs | ● | ● | ● | ● | ● | ● | ● | ● |
| Monitor (CSM-1901) recording |  | ●a |  | ●a | ●a | ●a | ●a |  |
| ECG monitor abnormality |  | ● |  |  |  |  |  |  |
| Height, weight and neck circumference | ● |  |  |  |  |  |  |  |
| ECOG-PS | ● |  |  |  |  |  |  |  |
| ASA classification | ● |  |  |  |  |  |  |  |
| Sleep apnea-related items | ● |  |  |  |  |  |  |  |
| 12-lead ECG | ● |  |  |  |  |  |  |  |
| Chest X-ray | ● |  |  |  |  |  |  |  |
| Ramsay sedation scale |  | ● | ● | ● | ● | ● | ● |  |
| Oxygen desaturation (<90%)  frequency and duration |  | ●b |  | ●b | | | |  |
| Oxygen desaturation (<95%) frequency |  | ●b |  | ●b | | | |  |
| Apnea (≥20 sec) frequency |  | ●b |  | ●b | | | |  |
| Oxygen desaturation (<90%)  oxygen desaturation (<90%) |  | ●b |  | ●b | | | |  |
| Oxygen desaturation (<95%)  oxygen desaturation (<95%) |  | ●b |  | ●b | | | |  |
| False alarm frequency |  | ●c |  | ●c | | | |  |
| Response to respiratory abnormality |  | ● |  |  |  |  |  |  |
| Technical trouble frequency |  | ●d |  |  |  |  |  |  |
| Position change frequency |  | ●d |  |  |  |  |  |  |
| BIS |  | ● |  |  |  |  |  |  |
| Sedative dose |  |  | ● |  |  |  |  |  |
| Coordination during treatment |  |  | ●e |  |  |  |  |  |
| Satisfaction with drug therapy |  |  |  |  |  |  |  | ●e |
| Support therapy |  | ● |  | ● | ● | ● | ● | ● |
| Observation of adverse events |  | ●f | ●f | ●f | ●f | ●f | ●f | ●f |

In some cases, data acquired prior to obtaining consent may be included in the study data. X-ray images and ECG data shall be acquired no more than 60 days prior to obtaining the consent of patients, and when no apparent heart disease or lung disease is observed, the use of such data is permitted and requires no retesting.

a: During treatment in the Safety Evaluation (Phase II), blood pressure, heart rate, SpO2, BIS and capnography/nasal pressure waveforms will be continuously recorded, while the doctor in charge of sedation monitors all items other than capnography waveforms. During the procedure that undergoes Usefulness Evaluation (Phase III), only one of capnography or nasal pressure will be recorded and displayed. After returning to the ward, SpO2 and capnography/nasal pressure waveforms (the same as those recorded during treatment) will be continuously recorded for a further three hours (one hour in the case of the usefulness evaluation).

b: During treatment the screens will be monitored and the state of respiration when SpO2 drops below 90% (frequency) and 95% (frequency) and oxygen desaturation (<90% and <95%) occurs will be recorded. In addition to the above, assistant investigators will analyze monitor records after the completion of the monitoring period and record the state of respiration when SpO_2_ drops below 90% (frequency and duration) and 95% (frequency), when oxygen saturation (<90% and <95%) occurs, and the frequency with which the patient experiences apnea (≥20 sec). Analysis of monitor records will be carried out for three hours after the completion of treatment for the Safety Evaluation (Phase II), and for one hour after the completion of the treatment for the Usefulness Evaluation (Phase III). (In the Safety Evaluation (Phase II), if respiratory abnormalities occur frequently after one hour has passed following the completion of the treatment, the duration of the observation period after the completion of the treatment for the usefulness evaluation will be reconsidered.)

c: In the post-treatment monitor analysis by the assistant investigators, if oxygen desaturation was not observed even without respiratory waveforms of more than three minutes, this will still be deemed to be a false alarm.

d: If technical trouble occurs, such as apnea or a respiratory abnormality being displayed due to a dislodged sensor, or if bodily movement occurs that requires two or more people to secure the safety of the patient or to continue the procedure, the doctor in charge of sedation will record such matters.

e: Operators will evaluate coordination during the treatment using VAS after the treatment has been completed. After confirming that the patient is fully awake after the treatment, on the same day, the degree of patient satisfaction regarding the treatment will be evaluated using VAS.

f: All events that occurred from the time of the commencement of the treatment will be checked to see if they equate to adverse events. All adverse events that occurred during the treatment period will be followed up during the post-treatment observation period. For details please see “8. Handling Adverse Events.”

g: If the study is discontinued, data that was scheduled to be obtained on the day following treatment will be collected, and no data will be collected after that using the testing equipment.

- - 1. Screening

The screening process will commence after consent has been obtained. The principal investigator or assistant investigators will carry out the following screening tests and select patients who satisfy the eligibility criteria and are not in conflict with the exclusion criteria. Items to check are shown below.

1. Patient background: sex, age, allergies, name of illness to be treated, history of illness, current medication, existing conditions, alcohol consumption, and history of smoking
2. Subjective symptoms
3. Vital signs: heart rate, blood pressure, temperature and SpO_2_
4. Height, weight and neck circumference
5. ECOG-PS
6. American Society of Anesthesiologists (ASA) Physical Status Classification
7. Sleep apnea-related items (STOP-Bang questionnaire, Mallampati score, and submental soft-tissue excess)
8. 12-lead ECG
9. Chest X-ray
   - 1. **Examination, Testing and Evaluation**
        1. **During Treatment**
10. Sedation start/stop times
11. Subjective and objective findings
12. Vital signs: blood pressure, mean blood pressure and heart rate.

Blood pressure will be taken at 5-minute intervals, other items will be taken at 2.5-minute intervals for 15 minutes after the start of sedation, and at 5-minute intervals thereafter. Measurements will also be taken as required at the discretion of the doctor in charge of sedation. Collect the data for the worst levels (highest or lowest).

1. Monitor (CSM-1901) data
2. Record any ECG monitor abnormalities.
3. SpO_2_: <90% (frequency and duration), <95% (frequency)
4. Apnea (≥20 sec) frequency
5. Respiratory status under SpO_2_ of <90% and <95%
6. False alarm frequency
7. Technical trouble and bodily movement frequency
8. Response to respiratory abnormality
9. BIS
10. Ramsay sedation scale

To be carried out after the commencement of sedation when the condition is stable, and to be reconsidered as required whenever the depth of sedation changes.

1. Support therapy
2. Observation of adverse events
   - - 1. **At the End of Treatment**
3. Subjective and objective findings
4. Vital signs: heart rate, blood pressure, temperature, SpO_2_ and respiration rate
5. Ramsay sedation scale
6. Sedative dose
7. Coordination during procedure (VAS)
8. Observation of adverse events
   - - 1. **After Procedure (30 min, 1 hr, 2 hrs and 3 hrs)**
9. Subjective and objective findings
10. Vital signs: heart rate, blood pressure, temperature, SpO_2_ and respiration rate
11. Monitor (CSM-1901) data
12. SpO_2_: <90% (frequency and duration), <95% (frequency)
13. Apnea (≥20 sec) frequency
14. Respiratory status under SpO_2_ of <90% and <95%
15. Ramsay sedation scale
16. Support therapy
17. Observation of adverse events
    - - 1. **Following Day**
18. Subjective and objective findings
19. Vital signs: heart rate, blood pressure, temperature and SpO_2_
20. Degree of satisfaction with treatment (VAS)
21. Support therapy
22. Observation of adverse events
23. **Handling Adverse Events**
    1. **Definition of Adverse Event**

An adverse event refers to an
undesired or unintentional sign (including abnormal fluctuations in clinical data), symptom or disease that appears after the commencement of the study, regardless of the causal relationship with the testing equipment.

- 1. **Definition of Disease, etc.**

Disease, etc., refers to an illness, disorder, death or infectious disease that is suspected to have occurred due to the carrying out of a particular clinical study, including abnormal clinical test data and other symptoms (referred to as adverse events that have an undeniable causal relationship with the particular clinical study). Moreover, if a defect is detected in a medical device used in the study and it is suspected that it may cause disease, etc., this will also be reported.

Matters regarding diseases, etc., that must be reported to the Authorized Clinical Study Review Board/Minister of Health, Labour and Welfare are determined in “9.3. Reporting Diseases, etc., and Defects.”

- 1. **Dealing with Participants when Adverse Events Occur**

When an adverse event is observed, the principal investigator or assistant investigators will immediately take appropriate measures, and if treatment is required they will inform the participant.

- 1. **Obtaining Information regarding Adverse Events**

With regard to all adverse events that occurred during the reporting period shown below, the principal investigator or assistant investigators will enter them in medical records and case report forms truthfully.

- 1. **Adverse Event Reporting Period**

All adverse events that occur between the first date on which the testing equipment is used and the day following the last day on which the testing equipment is used will be collected regardless of the causal relationship. Adverse events that are deemed to have a causal relationship with the testing equipment will be observed until such adverse events disappear or the symptoms stabilize.

All serious adverse events that occur before seven days after the last day on which the testing equipment is used will be reported, and such adverse events will be observed until they disappear or the symptoms stabilize. Adverse events that are deemed not to have any relationship with the testing equipment will be observed until they disappear or for 28 days after the completion (discontinuation) of the study.

The principal investigator or assistant investigators will enter all of the adverse events that occurred during the reporting period shown above in medical records and case report forms truthfully.

- 1. **Reporting Details Required for Evaluating Adverse Events**

1. Name of the adverse event.
2. Date on which the event occurred.
3. Date on which the outcome was decided.
4. Outcome: recovered (or disappeared), alleviated, recovered or disappeared with aftereffects, yet to recover, deceased, or unknown.
5. Procedure (use of testing equipment): as scheduled, discontinued, or not-applicable.
6. Other procedures: none, drug therapy, or other.
7. Severity: Not severe or severe.
8. Symptom evaluation: CTCAE (Ver. 4.0) Grade 1, 2, 3, 4 or 5.
9. Causal relationship with testing equipment: Yes, no.
10. Predictability: Known or unknown
    - 1. **Adverse Event Recoverability and Causal Relationship with Testing Equipment**

The disappearance of an adverse event refers to the state of the patient without the adverse event in question or the recovery of a patient to the state prior to the use of the equipment. The causal relationship between an adverse event and the testing equipment will be determined in light of the general condition of the patient, complications, concomitant drugs/therapies, and time factors. When it is unknown whether there is causal relationship between an adverse event and the testing equipment, such cases will be entered as “Yes” under Causal Relationship.

- - 1. **Adverse Event Predictability Evaluation**

The principal investigator will refer to the document that shows the latest overview of the testing equipment (package insert/operation manual, etc.) and determine the predictability of adverse events. Adverse events for which it is impossible to predict occurrence tendencies from the summary documents for such equipment, such as the number of instances of the adverse event occurring, or the frequency of occurrence or prerequisites for the appearance of a particular adverse event, shall be entered as “unknown” and where they can be predicted they shall be entered as “known.” As there is no existing summary document for the nasal pressure monitor, the package insert for a portable sleep apnea tester will be used for reference.

- 1. **Expected Adverse Events**
     1. **Expected Adverse Events Associated with ERCP, EUSFNA, ESD and DBE**

Expected adverse events associated with ERCP, EUSFNA, ESD and DBE include acute pancreatitis, acute cholangitis, bleeding, infection, gastrointestinal perforation, peritonitis and aspiration pneumonia.

- - 1. **Adverse Events due to Propofol**

Potential adverse events caused by propofol include hypotension, anaphylactoid symptoms, bronchospasm, pharyngeal obstruction, transient apnea, epileptic-like movements, bradycardia, ventricular extrasystole, left bundle branch block, pulmonary edema, delayed emergence, and rhabdomyolysis.

- - 1. **Adverse Events due to Fentanyl**

Potential adverse events caused by fentanyl include drug dependence, respiratory depression, respiratory distress, blood pressure reduction, shock, arrhythmia, agitation, and cyanosis.

1. **Handling Serious Adverse Events**
   1. **Definition of Serious Adverse Event**

A serious adverse event is defined as one that corresponds to any of the following.

(1) Death

(2) An event that may lead to death.

(3) Impairment (the appearance of functional failure of such a degree that it hinders daily life).

(4) An event that may lead to impairment.

(5) An event that requires hospitalization or an extension of hospitalization in a hospital or clinic for treatment.

(6) A serious event similar to the cases described in (1) to (5).

(7) A congenital disease or abnormality.

Note: The “hospitalization’ mentioned in (5) does not include hospitalization or extension of hospitalization for retesting, follow-up testing or treatment, or hospitalization for the purpose of treatment or testing that was already scheduled to be performed during the timeframe of the study (scheduled surgery and testing). (However, events that newly occur during such hospitalization will be handled as an adverse event.)

- 1. **Serious Adverse Events Subject to Reporting**

All serious adverse events that occur during the study, and other serious adverse events that are suspected to have occurred due to testing equipment after the completion (discontinuation) of the study, will be reported.

Acute cardiorespiratory failure that occurs during the period in which adverse events are observed within the study period (from the commencement of sedation to the following day) will be defined as follows in accordance with CTCAE. Adverse events classed as Grade 3 or above will be reported as serious adverse events regardless of the causal relationship with the testing equipment.

| Grade 3 | Events that are not life-threatening, but which require general management using tracheal intubation or general anesthesia even after the measures described in “5.7. Response to Respiratory Abnormalities” as defined in the Protocol have been taken. |
| --- | --- |
| Grade 4 | Events that require resuscitation using cardiac massage and tracheal intubation due to deterioration in cardiovascular dynamics. |
| Grade 5 | Death. |

- 1. **Reporting Disease, etc., and Defects**

When the principal investigator comes to the knowledge regarding the disease/condition that are listed in the table below during the execution of the specific clinical study, they will report within the specified period.

**Table: Reporting Diseases, etc. (For Specific Clinical Study involving Medical Devices that are Unapproved or Not Listed for Use under National Health Insurance)**

|  |  |  | Report to the Pharmaceuticals and Medical Devices Agency (PMDA) | Report to Clinical Study Review Board |
| --- | --- | --- | --- | --- |
| Disease, etc. | Unknown | Death | 7 days | 7 days |
|  |  | Serious | 15 days | 15 days |
|  |  | Non-serious |  | Regularly |
|  | Known | Death |  | 15 days |
|  |  | Serious |  | Regularly |
|  |  | Non-serious |  | Regularly |

If a defect is found in the testing equipment used in the study that may cause a serious disease, etc., after reporting the matter to the participating institution within 30 days of coming to know about it, the principal investigator will report it to the Approved Clinical Study Review Board. Reporting procedures shall be in accordance with “9.4. Serious Adverse Event (disease, etc.,/defect) Reporting Procedure.”

**Table: Reporting Defects in Specific Clinical Studies involving Medical Devices that are Unapproved or Not Listed for Use under National Health Insurance**

|  |  |  | Report to the Pharmaceuticals and Medical Devices Agency (PMDA) | Report to Clinical Study Review Board |
| --- | --- | --- | --- | --- |
| Defect  (medical device) | Unknown | Risk of death |  | 30 days |
|  |  | Risk of serious adverse event |  | 30 days |
|  |  | Risk of non-serious event |  |  |
|  | Known | Risk of death |  | 30 days |
|  |  | Risk of serious adverse event |  | 30 days |
|  |  | Risk of non-serious adverse event |  |  |

- 1. **Serious Adverse Event (Disease, etc./Defect) Reporting Procedure**

In cases where an adverse event occurs and the principal investigator determines that it is serious, information regarding the adverse events will be handled in accordance with the following procedure.

1. Report from the principal investigator to the head of the participating institution

The principal investigator reports to the head of the participating institution regarding information concerning the adverse event regardless of the causal relationship as promptly as possible. First an Emergency Report (Report 1) is sent, and then a Detailed Report (Report 2) will follow.

1. Report to the Approved Clinical Study Review Board/Minister of Health, Labour and Welfare

In accordance with the Clinical Trials Act, in cases where the principal investigator determines that reporting to the Approved Clinical Study Review Board/Minister of Health, Labour and Welfare is required as described in “9.3. Reporting Diseases, etc. and Defects,” the principal investigator will prepare a Medical Device Diseases, etc., and Defect Report (Standard Form 9) and a Diseases, etc., Report (Medical Device) (Annex Form 2-2), and submit them to the Approved Clinical Study Review Board/Minister of Health, Labour and Welfare. Regular reports regarding diseases, etc., will be made to the Approved Clinical Study Review Board using the Regular Report Form (Standard Form 5).

1. Information Shared with Manufacturers/Retailers of Testing Equipment

When making 7-day or 15-day reports regard diseases, etc., such information will also be shared with manufacturers/retailers of testing equipment at the same time.

1. Handling Additional Information

When the principal investigator of the participating institution where the adverse event in question occurred acquires additional information regarding the adverse event, an additional report will be made to the head of the participating institution as promptly as possible. Procedures for handling additional information will be in accordance with 2) to 4) of this section.

1. Response based on the Pharmaceutical and Medical Device Safety Information Reporting System.

Post-marketing drugs will be handled based on the Pharmaceutical and Medical Device Safety Information Reporting System, and reports will be submitted to the Ministry of Health, Labour and Welfare as required.

- 1. **Regular Reports**

Regular reports will be submitted to the Approved Clinical Study Review Board and the Minister of Health, Labour and Welfare regarding diseases, etc., in accordance with the Clinical Trials Act. Regular reports will be submitted to the Approved Clinical Study Review Board as a rule each year after one year from the day on which the study plan was submitted to the Minister of Health, Labour and Welfare, within two months of the completion of such period. Reports to the Minister of Health, Labour and Welfare will be made within one month of the day on which the Approved Clinical Study Review Board expressed their opinion.

1. **Evaluation Items**
   1. **Primary Endpoint**

- Hypoxemia occurrence rate (SpO_2_ <90%): number of cases / total number of eligible participants

Basis for Primary Endpoint Selection

This particular clinical study will be conducted to verify that there is no difference in the frequency of hypoxemia between the nasal pressure monitor group and the capnography group. SpO_2_<90% will be used as the general index for hypoxemia.

- 1. **Secondary Endpoints**

**Secondary Endpoints for Usefulness**

- Frequency of apnea (≥20 seconds)
- Degree of participant satisfaction (VAS)
- Coordination during procedure (VAS)

**Secondary Endpoints for Safety**

Adverse Event Occurrence Rate

NCI-Common Terminology Criteria for Adverse Events v4.0 (CTCAE Ver. 4.0) JCOG version will be used to evaluate adverse events.

Basis for Secondary Endpoint Selection for Usefulness

Items where a difference in occurrence frequency between the capnography group and the nasal pressure monitor group is considered to be important will be evaluated as secondary endpoints.

Basis for Secondary Endpoint Selection for Safety

General evaluation standards will be used.

- 1. **Exploratory Endpoints**
- Comparison with capnography waveforms.
- Waveform analysis at the time of respiratory abnormality.
- Consideration of respiratory abnormality-related risk factors (including obesity, age, history of smoking, drug administration dose, treatment duration, and SAS).

Basis for Exploratory Endpoint Selection

There is a possibility that nasal pressure monitoring can detect the minute changes in waveforms that appear prior to the appearance of a respiratory abnormality. We will compare the changes in the waveforms prior to any drop in SpO_2_ between the capnography group and the nasal pressure monitor group, and analyze to what degree the monitor observer is able to detect changes in waveforms. As there is also a possibility that nasal pressure monitoring can accurately detect the patterns for respiratory depression and obstructive ventilatory impairment, we will analyze the frequency of those events in both groups. We will also consider the relationship between respiratory abnormalities (hypoxemia, apnea and obstructive ventilatory impairment) and obesity, age, drug dose, procedure duration and SAS in order to identify factors relating to respiratory abnormalities.

1. **Statistical Considerations**

The statistical analysis plan overview for the clinical study is shown below. Statistical analysis plan details are contained in the Statistical Analysis Plan. Although the overview of the Study Protocol for the clinical study may be revised in the Statistical Analysis Plan, in cases where the primary endpoint definition or analysis method is to be changed, the Study Protocol will be revised.

- 1. **Analysis Population**
     1. **Full Analysis Set (FAS)**

The FAS will be all registered participants whose usefulness data is entered after randomization. However, those of them whose baseline data was not available and those who are seriously in bleach of the Study Protocol (no consent, registered outside the agreed period) will be excluded.

- - 1. **Per protocol set (PPS)**

PPS will be the population of FAS minus those cases that are seriously in bleach of the Study Protocol, in terms of research method or concurrent therapy.

1. Selection criteria violation
2. Exclusion criteria violation
3. Contraindicated drug violation
4. Contraindicated therapy violation
   - 1. **Safety analysis set (SAS)**

SAS will the registered patients who were fitted with the testing equipment (the monitor) at least once and underwent endoscopy under sedation.

- 1. **Target Number of Cases and Basis for Selection**

**● Safety Evaluation (Phase II)**

In calculating the target number of participants for the safety evaluation cohort, with the expected value of 20% for hypoxemia (<90%), threshold at 40%, significance level of 0.05 on both sides and statistical power of 80%, required number is 43 cases. In consideration of drop-out, we set the target at 45 cases.

**● Usefulness Evaluation (Phase III)**

The purpose of the usefulness evaluation is to prove that there is no difference in the incidence rate of hypoxemia (SpO2 <90%) between nasal pressure monitor group and capnography group, meaning that nasal pressure monitor is not inferior to capnography. According to reports, the incidence rate of declined SpO_2_ to under 90% in the endoscopic procedure under sedation is 12–47% when propofol is used as sedative,[4, 8, 9, 10] With regard to required number of registered patients, calculation based on 20% hypoxemia incidence rate, with non-inferiority margin of 15% for nasal pressure monitor against capnography, statistical power of 80% and significance level of 0.05 on both sides results in 224 patients for both groups (112 patients respectively).

- 1. **Handling Case Records**

In principle, the principal investigator and statistic specialists will decide on how the information on cases will be handled. In principle, the principal investigator and a statistic specialist will decide on how the information on cases will be handled.

- 1. **Handling Data**

When computing or analyzing data, as a rule, the data will be handled as shown below. If a question arises, it will be resolved through discussion between a statistic specialist and the study coordinator.

Missing data will not be supplemented. As sensitivity analysis, mixed-effects model for repeated measures, which compensates for data mission, will be used. Details will be contained in the Statistical Analysis Plan.

- 1. **Statistical Analysis Items and Plan**

● Safety Evaluation (Phase II)

Carry out analysis after the data is fixed following the completion of study involving all cases that are the subjects of safety evaluation. In the usefulness evaluation of all participants, the primary analysis will be the analysis of the FAS, and analysis of the PPS that complies with the Study Protocol will be carried out as a reference. Safety analysis will be carried out with the SAS.

Details of the statistical analysis will be determined in the Statistical Analysis Plan, which will be prepared elsewhere before the data is locked.

● Usefulness Evaluation (Phase III)

Data from the usefulness evaluation will be analyzed after it is fixed following the completion of the study that involves all cases. In the usefulness evaluation of all participants, the primary analysis will be the analysis of the FAS, and analysis of the PPS that complies with the Study Protocol will be carried out as a reference. Safety analysis will be carried out with the SAS.

Details of the statistical analysis will be determined in the Statistical Analysis Plan, which will be prepared elsewhere before the data is locked.

- - 1. **Patient Background Analysis**

Patient data distribution and summary statistics will be obtained for each group by population. With regard to nominal variables, frequency of the category and proportion will be shown for each group. With regard to metric variables, summary statistics (number of cases, mean value, standard deviation, minimum value, median and maximum value) will be obtained for each group. In the comparison between two groups, Pearson's chi-square test will be used for nominal variables, but if the cells with expected frequency <5 account for 20% or more, Fisher’s exact test will be used. The t-test will be used for metric variables. Significance level will be 0.05 on both sides.

- - 1. **Usefulness Analysis**
       1. **Primary Analysis**

The main purpose of the study is to verify using the incidence rate of hypoxemia as primary endpoint that nasal pressure monitor is not inferior to capnography. Frequency and proportion in each group will be collated and **two-sided 95% confidence interval will be computed** When the upper limit of the 95% confidence interval in the comparison of hypoxemia incidence rate goes 15% lower than the non-inferiority margin, we deem that non-inferiority was proven.

To verify the null hypothesis that incidence rate of hypoxemia in both groups is the same in the primary analysis, Mantel–Haenszel test will be applied to the data stratified using assignment factors. Significance level will be 0.05 on both sides.

- - - 1. **Secondary Analysis**

Secondary analysis regarding usefulness will be carried out for the purpose of giving supplementary consideration to the results of primary analysis. No multiplicity adjustment will be carried out in the secondary endpoints analysis regarding usefulness .

Frequency of apnea (≥20 sec), degree of patients’ satisfaction and coordination during procedure will be evaluated with capnography group and nasal pressure monitor group, and the difference between those two groups will be analyzed using Pearson’s chi-square test. Significance level will be 0.05 on both sides, and confidence interval, and **two-sided 95% confidence interval will be computed.**

- - 1. **Safety Analysis**

Frequency of adverse event occurrence is the evaluation items to consider safety, therefore data table will be created. To estimate the proportion, exact 95% confidence interval for binomial distribution will computed for each group. Inter-group comparison will be carried out as required using Fisher's exact test.

- - 1. **Intermediate Analysis**

Data will be locked when registration of all cases for the Safety Evaluation (Phase II) is completed. The fixed analysis results will be disclosed to the independent data-monitoring committee (IDMC) (see 11.6) that oversees the study. The IDMC will evaluate the fixed data, and when they find the following situation, the study will be discontinued without carrying out registration for the usefulness evaluation.

- Rate of discontinued cases due to default accounted for 20% or more.
- Hypoxemia (SpO_2_<90%) was found in 60% or more of the cases.
- The IDMC determined that it will be inappropriate to conduct usefulness evaluation.

When the IDMC verified the safety, the participants will be registered for the usefulness evaluation. No intermediate analysis will be carried out in the usefulness evaluation.

- 1. **Independent Data-Monitoring Committee (IDMC)**

The independent data-monitoring committee (IDMC) will be established for this study. The IDMC will be independent of the people who conduct the study, and consist of two or more specialists whose position have no conflict of interest with this particular clinical study. The IDMC will provide appropriate advice and guidance in order to secure the safety of the participants and ethical and scientific soundness, based on the data monitoring protocol that is determined elsewhere for the purpose of securing safety of the participants. In cases where serious adverse events appear in this clinical study, the principal investigator may request the IDMC to deliberate concerning whether or not the study should be continued or revision of the Study Protocol. The IDMC will serve in writing the results of the deliberation to the principal investigator.

If the principal investigator determines in light of such results that it immediately requires emergency response due to urgency and influence of the situation, registration can be temporarily stopped, notification can be sent to related personnel and other similar measures can be taken.

- 1. **Final Analysis**

After the completion of follow-up period, case data will be fixed and analysis will be carried out. Analysis officer will prepare the Analysis Report and submit it to the study coordinator and the principal investigator.

1. **Compliance with Study Protocol and Nonconformity Management**
2. The principal investigator and assistant investigators will adhere to the Study Protocol and conduct the clinical study in order to avoid urgent risk for the participants, unless there are inevitable medical reasons.
3. Nonconformity in accordance with the Clinical Trials Act means incompliance with regulations, study protocol, manual, etc. and falsification and fabrication of research data. Deviation from the Study Protocol is also included in nonconformity.
4. The principal investigator or assistant investigators will record all nonconformities regardless of the reason.
5. When the principal investigator finds nonconformity, he/she will report it to the manager of the participating institution. When assistant investigators find nonconformity, they will promptly report it to the principal investigator. However, if it is a minor deviation that is acceptable based on the Study Protocol, the principal investigator and assistant investigators will decide the need for reporting to the manager of the participating institution.
6. When the principal investigator finds an especially serious nonconformity, he/she will promptly report to the Approved Clinical Study Review Board. Measures to prevent recurrence of such situation will be taken and assistant investigators will be informed of such situation. Serious nonconformity under such situation means those events that influence the human rights and safety of the participants of the study, progress of the study and its confidence, for example, serious breach of eligibility/exclusion criteria or discontinuation rules.
7. **Changes regarding Study Protocol, Case Report or Analysis Plan**
   1. **Revising Study Protocol and Case Report**
8. The person who oversees the study, when it is deemed necessary, will provide a proposal to revise the Study Protocol or case report to the principal investigator together with latest annexed document and other necessary reference materials and information.
9. The research administrator will give sufficient consideration to the materials and information mentioned in the previous paragraph that he/she provides to the principal investigator, and allow adequate time for the principal investigator to consider the matters.
10. The principal investigator will promptly submit the revised Study Protocol or case report to the head of the participating institution and promptly acquire approval of the Approved Clinical Study Review Board
11. If it is determined that changes have to be made to the Study Protocol that was submitted to the Minister of Health, Labour and Welfare, a notification regarding such changes will be submitted.
    1. **Revising Analysis Plan**

When the content of the Analysis Plan is revised, the statistics specialist will enter all changes in the Analysis Report for the study. A record will be kept of all such changes to the Analysis Plan.

1. **Cancellation, Discontinuation or Completion of the Study**
   1. **Standard for Canceling or Discontinuing the Study**

If the information described in the following paragraphs is revealed and it is thought that continuing with the study may be difficult, the research administrator will discuss whether to cancel or discontinue the study with the principal investigator and decide on the appropriate course of action.

1. Appearance of unexpected serious adverse events.
2. Information suggesting that it will be impossible to forecast the tendency for expected serious adverse events to occur, including the number of cases or the incidence rate and the conditions under which they appear, based on the results of the questionnaire and the package insert.
3. Cases where information is revealed which shows that some of the serious adverse events, which were previously thought to have no causal relationship, actually have an undeniable causal relationship in light of the occurrence tendency revealed in the data, such as number of cases, the frequency, and the conditions under which they occur.
4. Study reports showing that the testing equipment defect occurrence tendency, including the number of cases, frequency and the conditions under which the defect occurs, has significantly changed.
5. Study reports showing that there is a risk of causing cancer or other serious illnesses, disorders, or death.
6. Information suggesting that the trial in question will not prove the usefulness of the testing equipment.
7. Information showing that the equipment has no benefit or effect on the illness that is the subject of the study.
8. In cases where information is acquired regarding measures that the authorities will take to prevent the occurrence of a health hazard and its spread, such as stopping the manufacture, importation or sale of medical devices that contain parts used in the testing equipment, collecting such devices, and/or disposing of them.
9. In cases where after the completion of the safety evaluation the IDMC determines that it is inappropriate to continue with the study.
   1. **Procedure to Cancel or Discontinue the Study**

In cases where the study will be cancelled or discontinued after discussion among the study investigators, the principal investigator will promptly inform the head of the participating institution in writing regarding the details and the reasons involved. The principal investigator will also inform eligible participants of the same, and take appropriate measures, such as changing treatments to a more appropriate type.

- 1. **Procedure for Cancelling or Discontinuing the Study at the Individual Institution**

In cases where the study is cancelled or discontinued, the principal investigator will promptly inform the head of the participating institution in writing regarding the details and the reasons involved.

- 1. **Completion of the Study**

After the completion of the study, the principal investigator will notify the head of the participating institution in writing regarding the fact that the study has been completed, and provide documents showing a summary of the results of the study.

1. **Data Management**
   1. **Data Entry Method and Management**
2. Submitting Case Report Forms (CRFs).

Submit all CRFs to the data center.

1. Enter all CRFs into the system.

The data center will use a system, with which it is possible to acquire access logs and validation, to enter the CRF data and create data sets.

1. Keep CRFs.

The original CRFs will be kept at the data center.

1. Database Lock

The database will be locked after it has been verified by the principal investigator.

- 1. **Identification of First-hand Entry into CRFs (Raw Data)**

In this clinical study, the following documents will be deemed original data (raw data).

1. Records concerning the consent of the participants and information provided to the participants.
2. Records that form the basis for CRFs, such as medical records, nursing records, clinical examination data, and scan images. Data stored in electronic medical records is also deemed raw data.
3. Monitoring records that were recorded in the testing equipment and then transferred to a hard drive.
4. Records concerning the use of testing equipment.
5. Documents or records that are essential to the study in light of the guiding principle of the clinical study.

The items shown below are, when entered in CRFs, regarded as raw data. However, when they are contained in medical records, such medical records are regarded as raw data.

1. The aim of concomitant drugs or therapy.
2. The degree of adverse events, the outcome (including the results at the time of follow-up examination), the severity, decisions regarding the causal relationship with this particular testing equipment, and the reasons for such decisions.
3. The reasons for participant dropout.
4. The comments of the principal investigator or assistant investigators.
5. **Storing Raw Data and Other Records**

Information concerning participants that is recorded in the monitor (CSM-1901) will be transferred using USB and stored in a hard drive. The hard drive, consent forms and CRFs will be securely kept under the supervision of the principal investigator.

The head of the research institution and the principal investigator must appropriately store essential documents regarding the research, etc. (including copies of application forms, notifications from the head of hospital, copies of various applications/reports, the participant identification code list, consent forms, copies of case reports and other documents/records that are required to guarantee the credibility of the data) until at least five (5) years have passed from the date on which the study was completed.

The locked data will also be appropriately managed at the Division of Biostatistics of Keio University Hospital Clinical and Translational Research Center.

1. **Direct Perusal of Raw Data**

The head of the participating institution and the principal investigator will guarantee that investigators from monitoring organizations, the audit committee, the Approved Clinical Study Review Board, or government authorities are able to see all records, including raw data. They will verify that the study is conducted appropriately and that the credibility of the data is sufficiently secured.

1. **Quality Control and Assurance in the Study**
   1. **Quality Control**

The study will be audited for the purpose of verifying that it is conducted in accordance with the Study Protocol and that data is accurately collected and stored. Audits will be carried out in accordance with the Guidelines regarding Clinical Study Monitoring and Audits. In the main audit, the data entered in the CRFs that is accumulated in the data center will be regularly monitored. Such processes will follow the auditing procedure determined elsewhere to manage quality control at the institution (onsite/offsite monitoring) according to the degree of risk involved.
Personnel involved in audits must report the result of such audits/monitoring to the principal investigator. They must not leak information that has been acquired through their involvement in the work without due reason, even after ceasing to be involved in the work.

- 1. **Quality Assurance**

No audit will be conducted to assure the quality of the clinical study.

1. **Ethics**

This clinical study will be carried out in compliance with the Clinical Trials Act and the Declaration of Helsinki.

1. **Confidentiality**

The principal investigator must verify that personal information of participants is kept confidential.

1. In CRFs, participants are identified only with a unique identification code.
2. Documents containing the personal information of the participants, such as consent forms, will be handled by assistant investigators as confidential documents.
3. **Approved Clinical Study Review Board**

Prior to the commencement of this clinical study, the Authorized Clinical Study Review Board of the participating institution will examine the ethical, scientific and medical appropriateness of the study. The clinical study will be conducted after obtaining the approval of the Approved Clinical Study Review Board. The Authorized Clinical Study Review Board will continue to review the performance of the study at least once a year.

1. **Expected Advantages/Disadvantages Arising from the Study**
   1. **Expected Advantages**

Among the testing equipment used in the clinical study, capnography has been approved for use in the treatments used in this clinical study and has also been listed for use under National Health Insurance. It has been used in normal clinical practice. Although the nasal pressure monitor has yet to be approved as a medical device, the ability to constantly measure and provide early detection of respiratory abnormalities has proven to be useful, meaning that it can bring benefits to clinical practice. However, as the cost of drugs and treatments during the study period is paid for by the participants with contributions from private insurance and national insurance, patients gain no medical or economical advantage over normal medical treatment by participating in the study.

- 1. **Expected Disadvantages**

As mentioned above, although the nasal pressure monitor has yet been to be approved, nocturnal polysomnography, which applies the same principle, is widely used in normal medical practice. As seen in 6.2. Important Findings regarding the Testing Equipment, there are no disadvantages for patients, however, eligible participants (see 3.) will be carefully considered in order to minimize risk. Furthermore, consideration will also be given to the method of study (see 5.) as shown below. ① Sedatives will be administered by an endoscopist who has been fully trained in sedation by an anesthesiologist. ② The strength of the sedatives during the procedure will be controlled between BIS 60 and 80 as shown on the monitor. ③ In cases where SpO_2_ is observed to have declined (<90), apnea lasts for ≥20 seconds, or a respiratory abnormality (≤8 bpm or abnormal waveform) accompanied by a decline in SpO_2_ (<95%) is observed during sedation, the doctor in change of sedation will report the respiratory abnormality to the endoscopy team, and measures will be taken to improve respiration.

In cases where a serious or unexpected adverse event appears, the principal investigator may, as required, request the deliberation of the IDMC. Such adverse events will be carefully considered and investigated in accordance with the Clinical Trials Act and other relevant regulation, and necessary measures will be taken.

1. **Compensation for Health Damage and Insurance**
   1. **Compensation for Health Damage**

With regard to health damage, the principal investigator etc., will endeavor to assist in the recovery of the patient in question and offer appropriate medical treatment. The cost of treating such health damage will be handled in accordance with the cost contribution arrangement of the patient in the specified clinical study, etc.

- 1. **Enrollment in Clinical Trial Insurance (Compensation Insurance)**

The principal investigator and assistant investigators will enroll in a clinical trial insurance (compensation insurance) in order to be able to perform their responsibilities in cases where it is necessary to deal with health damage.

- 1. **Enrollment in Liability Insurance**

In addition to compensation insurance, the principal investigator and assistant investigators will enroll in liability insurance.

1. **Monetary Payments**

Costs for patients will not increase by participating in the study. Examination, treatment and other procedures relating to the primary condition and other complications will be provided within the scope of the coverage provided under National Health Insurance.

1. **Conflicts of Interest and Research Funds**

This clinical study will be conducted with funding from the Treatment Section of the Department of Gastroenterology and/or the hospital. No funds or benefits, etc., will be provided by Nihon Kohden Corporation, which is the manufacturer and retailer of the testing equipment, and researchers will plan and conduct the study independent of the company. The testing equipment will be loaned from Nihon Kohden Corporation at no cost.

This research (study) will be fairly conducted by the chief researcher (principal investigator) and assistant researchers (assistant investigators) of Chiba University. Research funds will be managed based on the Fund Plan. In terms of conflicts of interest in relation to this study, all interests will be appropriately managed in accordance with the Policy regarding Conflicts of Interest Associated with Clinical Studies after obtaining the approval of the Conflict of Interest Committee of Chiba University Hospital. The transparency of the study with regard to conflicts of interest will be maintained through regular reporting of the course of the research (trial) to the Conflict of Interest Committee. There are no conflicts of interest that should be disclosed to the principal investigator and assistant investigators for this clinical study. Prof. Shiro Isono of the Department of Anesthesiology, Pain and Palliative Care Medicine is one of the inventors of the measuring technology which formed the basis for the development of the testing equipment, and the patent for such technology is held by Nihon Kohden Corporation and Chiba University (Respiratory Status Screening Device: Patent Application No. 6204086 and 6075972).

1. **Disclosure of Information regarding the Study**

The principal investigator will enter a summary of the study in the Clinical Trial Study Protocol/Research Outline Disclosure System (http://jrct.niph.go.jp/) prior to the commencement of the study, and update the Study Protocol as required based on the revision and progress of the study. Documents to be disclosed will be the Study Protocol, the Audit Plan, the Statistical Analysis Plan, and the General Report. When the clinical study is completed, the results of the study will be entered without delay.

1. **Announcement of Results**
   1. **Announcement Method**

The principal investigator will announce the results of the study without delay when the study has been completed . When doing so, necessary measures will be taken to protect the human rights, other rights and benefits of researchers and related personnel. Methods of announcement will include presentation at academic conferences, publication in journals, and entry on public databases.

- 1. **Rules for Announcement**

The main thesis for publication will be submitted to an English language journal. Public release of the study without the results of endpoint analysis by the research administrator or the principal investigator by means of presentation at academic meetings or submission as a general article for the purpose of introducing the study, or by announcing patient background distribution or safety data at an academic meeting or by means of submission to a journal after the completion of registration may be carried out after obtaining the approval of the principal investigator. Announcement of other contents that do not include the main analysis and/or final analysis will not be carried out, with the exception of cases in which the approval of the IDMC has been obtained. In principle, the first author who announces the thesis containing the main research results (primary endpoint analysis results) will be decided through discussion, and the final author will be the principal investigator, with co-authors who contributed greatly to the study, based on the restrictions of the journal to which the thesis will be submitted. The principal investigator will decide whether personnel from the research office should be included in the list of authors according to the degree of their contribution. Authors who will release a minor thesis other than the main one (e.g. a report regarding secondary endpoints, a thesis regarding secondary analysis) will be selected by the research administrator. All co-authors will review the content of the thesis before submission and consent to the content. In cases where a co-author candidate does not agree with the content even after discussion, the research administrator may exclude such person from the co-authors.

The dominant presenter for the main presentation at an academic meeting (the first presentation of the primary endpoint) will be decided through discussion. As they may have to present the thesis at academic meetings multiple times, the investigators of the study will take turns to be a presenter. Presenters will be decided after obtaining the approval of the research administrator. However, the principal investigator will be responsible for the preparation and the contents of the presentation, and, as a rule, the study office or the principal investigator will liaise with the statistics specialists. Presenters other than the principal investigator may not receive the results of computation or analysis directly from the data center without the approval of the principal investigator, the statistics specialists, and the data center.

1. **Framework of Study**

as per the attached document

1. **References**

1) Beitz A, Riphaus A, Meining A, Kronshage T, Geist C, Wagenpfeil S, Weber A, Jung A, Bajbouj M, Pox C, Schneider G, Schmid RM, W

ehrmann T, von Delius S. Capnographic monitoring reduces the incidence of arterial oxygen desaturation and hypoxemia during propofol sedation for colonoscopy: a randomized, controlled study (ColoCap Study). Am J Gastroenterol. 2012 Aug;107(8):1205-12. doi: 10.1038/ajg.2012.136. Epub 2012 May 29.

2) Vargo JJ, Zuccaro G Jr, Dumot JA, Conwell DL, Morrow JB, Shay SS. Automated graphic assessment of respiratory activity is superior to pulse oximetry and visual assessment for the detection of early respiratory depression during therapeutic upper endoscopy. Gastrointest Endosc. 2002 Jun;55(7):826-31.

3)　Rhodri Saunders, Michel MRF Struys, Richard F Pollock, Michael Mestek, Jenifer R Lightdale. Patient safety during proceduralsedation using capnography monitoring: a systematic review and meta-analysis. BMJ open. 2017.7, 2016-013402

4）Beitz, A.Riphaus, A.Meining, A.Kronshage, T.Geist, C.Wagenpfeil, S.Weber, A.Jung, A.Bajbouj, M.Pox, C.Schneider, G.Schmid, R. M.Wehrmann, T.von Delius, S. Capnographic monitoring reduces the incidence of arterial oxygen desaturation and hypoxemia during propofol sedation for colonoscopy: a randomized, controlled study (ColoCap Study) Am J Gastroenterol 2012; 107:1205–1212;

5）American Academy of Sleep Medicine：International Classification of Sleep Disorders．Third Edition（ICSD－3），Darien，IL：American Academy of Sleep Medicine，2014；63－68．

6) Ryuma Urahama, Masaya Uesato, Mizuho Aikawa, Yukiko Yamaguchi, Koichi Hayano, Tomoaki Matsumura, Makoto Arai, Reiko Kunii, Shiroh Isono, Hisahiro Matsubara. Polysomnographic assessment of respiratory disturbance during deep propofol sedation for endoscopic submucosal dissection of gastric tumors. World J Gastrointest Endosc 2018 November 16; 10(11): 340-347

7) Kohzuka Y, Isono S, Ohara S, Kawabata K, Kitamura A, Suzuki T, Almeida FR, Sato Y, Iijima T. Nasopharyngeal Tube Effects on Breathing during Sedation for Dental Procedures: A Randomized Controlled Trial. Anesthesiology. 2019 Jun;130(6):946-957

8) Friedrich-Rust M, Welte M, Welte C, Albert J, Meckbach Y, Herrmann E, Kannengiesser M, Trojan J, Filmann N, Schroeter H, Zeuzem S, Bojunga J. Capnographic monitoring of propofol-based sedation during colonoscopy. Endoscopy. 2014 Mar;46(3):236-44.

9)　Peter Klare, Johanna Reiter, Alexander Meining, StefanWagenpfeil, Tim Kronshage, Christoph Geist,Stefan Heringlake, Christoph Schlag,Monther Bajbouj, Gerhard Schneider, Roland M. Schmid, TillWehrm Stefan von Delius,, Andrea Riphaus

10) Riphaus A, Wehrmann T, Kronshage T, Geist C, Pox CP, Heringlake S, Schmiegel W, Beitz A, Meining A, Müller M, von Delius S. Clinical value of the Integrated Pulmonary Index® during sedation for interventional upper GI-endoscopy: A randomized, prospective tri-center study. Dig Liver Dis. 2017 Jan;49(1):45-49. Capnographic monitoring of midazolam and propofolsedation during ERCP: a randomized controlled study (EndoBreath Study) Endoscopy. 2016 Jan;48(1):42-50.
